# Region-specific weighting of sensory intensity and reward prediction error by dopamine signals

**DOI:** 10.1016/j.isci.2026.117130

**Published:** 2026-08-14

**Authors:** Sarah-Julie Bouchard, Joël Boutin, Martin Lévesque, Vincent Breton-Provencher

**Affiliations:** 1 CERVO Brain Research Centre, Québec City, QC G1J 2G3, Canada; 2 Department of Psychiatry and Neuroscience, Université Laval, Québec City, QC G1V 0A6, Canada

**Keywords:** dopamine, reward prediction error, classical conditioning, striatum, nucleus accumbens, prefrontal cortex, amygdala, dopamine sensors, optogenetics

## Abstract

Growing evidence shows that dopamine signaling is diverse, with dopaminergic neurons responding to rewarding and aversive stimuli. Here, we hypothesized that this heterogeneity arises from a distributed balance between two components of dopamine signaling: sensory intensity of a stimulus, and reward prediction error. To test this, we simultaneously recorded dopamine release across striatal and cortico-amygdalar circuits using multi-site fiber photometry and a fluorescent dopamine sensor during a classical conditioning paradigm. Using a regression model, we found that striatal areas such as the dorsal striatum and nucleus accumbens emphasized reward prediction error, whereas the medial prefrontal cortex and basolateral amygdala favored sensory intensity. Noise correlation analyses further supported these findings by revealing two modules of coordinated dopamine activity: one linking striatal regions and another linking cortical-amygdalar circuits. Together, our results suggest that dopamine balances sensory and reward information, a mechanism that may support attentional processes alongside its role in associative learning.

## Highlights

• Multi-site dopamine recordings reveal region-specific sensory and reward encoding
• Striatal dopamine primarily encodes reward prediction error over sensory intensity
• Cortical and amygdalar dopamine preferentially encodes sensory intensity
• Striatal and cortico-amygdalar circuits form distinct modules of dopamine signaling

## Graphical abstract

**Figure:**
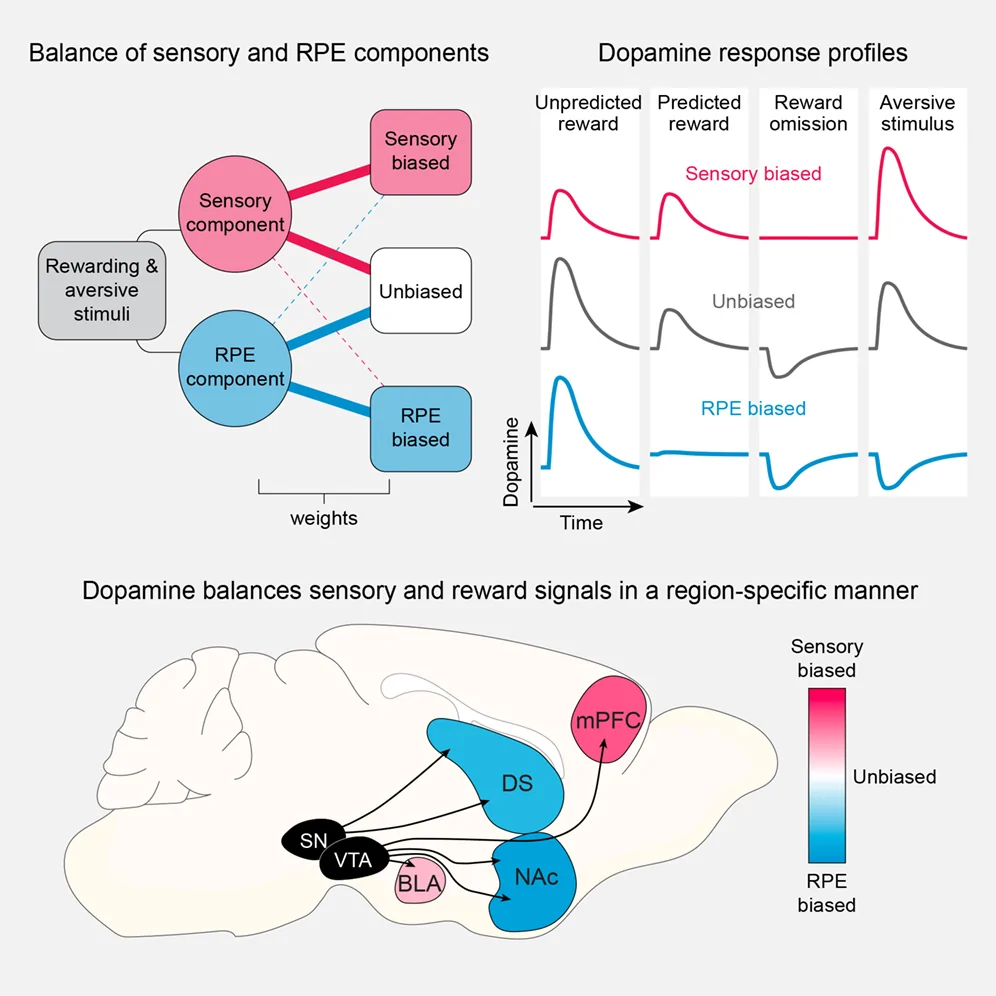

## Teaser

Neuroscience; Sensory neuroscience; Molecular interaction

## Introduction

The dopaminergic system is critical for goal-directed and reinforcement learning. Dopaminergic neurons in the midbrain encode reward prediction error (RPE) to downstream brain regions such as the striatum. This RPE signal is characterized by transient increases or decreases in activity following outcomes that are better or worse than expected, respectively,^1^^,^^2^^,^^3^ updating future behavioral choices. Consistent with this role in reinforcement learning, activating dopamine release promotes reward-seeking behavior and can reinforce actions similarly to actual reward delivery,^4^^,^^5^^,^^6^^,^^7^^,^^8^ whereas blocking dopamine activity disrupts cue-reward associations.^9^^,^^10^

Numerous studies have also shown that a subset of dopamine neurons responds to salient stimuli,^11^^,^^12^^,^^13^^,^^14^ or to non-reward-related events.^15^^,^^16^ Although individual dopamine neurons can encode non-reward-related behavioral variables, they often simultaneously encode RPE, suggesting that dopamine signaling may multiplex other signals with reward.^16^^,^^17^ Importantly, these non-reward dopamine signals are also observed at the level of dopamine release in downstream target regions. Dopamine responses to non-rewarding events have been reported in parts of the dorsal striatum (DS)^18^^,^^19^ and the tail of the striatum,^20^^,^^21^^,^^22^ subregions of the nucleus accumbens (NAc),^23^^,^^24^^,^^25^^,^^26^^,^^27^ the medial prefrontal cortex (mPFC),^28^^,^^29^ and the basolateral amygdala (BLA).^29^^,^^30^^,^^31^

To explain these seemingly non-reward related signals, a theory has emerged proposing that dopamine neurons encode two distinct and sequential components^32^: First, a short-latency component, which precedes the time at which reward value could be processed, reflects the initial detection of an event (sensory intensity).^33^ Second, a longer-latency component reflects the motivational significance of an event, also known as RPE. At the level of neuronal spiking, these components can be dissociated by comparing the later value-dependent responses with the early transient increase in activity that is largely independent of reinforcement valence,^34^ or reward size.^35^^,^^36^ However, this unifying framework has only been examined in single target regions and not been systematically tested at the level of dopamine release across distinct target regions, which is crucial considering the substantial heterogeneity observed across dopaminergic projections. As a result, it remains unclear how sensory intensity and RPE encoding by dopamine is distributed across target brain regions to shape learning and behavior.

Here, we used multi-site fiber photometry recordings of the fluorescent dopamine sensor GRAB-DA^25^ to simultaneously measure population-level biases in dopamine signals across striatal and non-striatal regions in response to stimuli varying in sensory intensity and RPE. Critically, all measurements were obtained within the same animals, allowing direct comparison of regional dopamine signaling while controlling for inter-individual variability in sensory sensitivity and learning. Using constrained least-squares regression to model dopamine release responses to distinct outcomes, we found that dopamine release in the mPFC and BLA is biased toward sensory intensity encoding, whereas release in the NAc and DS is biased toward RPE. Together, these findings reconcile divergent observations of dopaminergic responses to reward and non-reward events by revealing a region-specific weighting of dopaminergic sensory and outcome signals, providing a unified framework for understanding dopamine’s role in learning and adaptive behavior.

## Results

### Dopamine dynamics across striatal and cortico-amygdalar circuits during diverse outcomes

**Figure 1:**
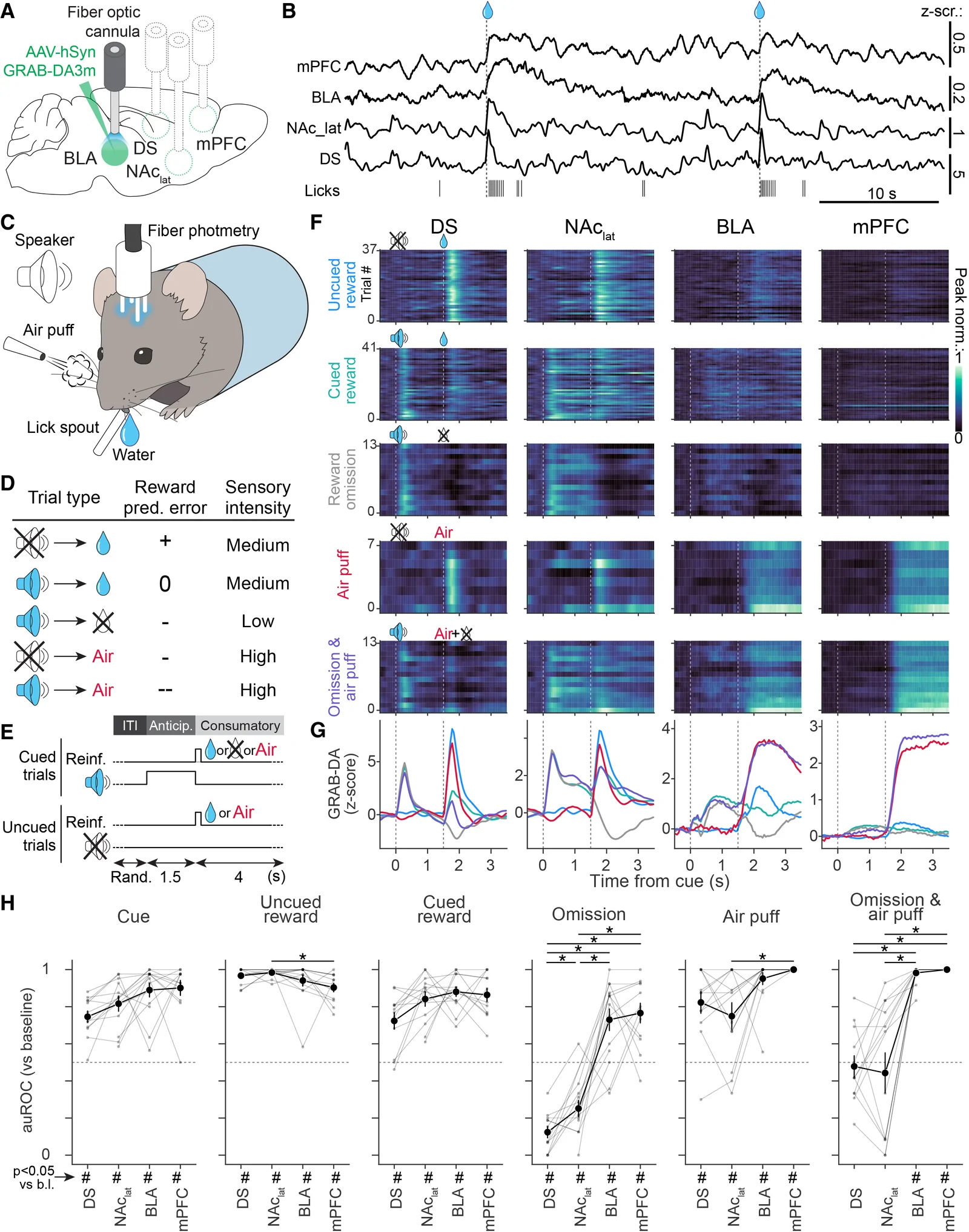
Dopamine dynamics across striatal and non-striatal regions during positive and negative outcomes (A) Surgical strategy for GRAB-DA3m expression and fiber optic implantation in dorsal striatum (DS), nucleus accumbens lateral shell (NAclat), basolateral amygdala (BLA), and medial prefrontal cortex (mPFC). (B) Example GRAB-DA fluorescence traces simultaneously recorded from mPFC, BLA, NAclat, and DS during uncued reward presentation. (C) Behavioral setup. (D) Trial structure and associated expectancy-dependent reward prediction error and sensory intensity. (E) Timing of inter-trial interval, tone, and reinforcement. (F) Raster plots of an example session showing GRAB-DA responses in four regions aligned to cue onset for each trial type. (G) Average GRAB-DA responses (mean ± SEM) for each trial type and region aligned to cue onset. Colors are the same as in F. (H) Area under the ROC (auROC) quantification of cue-evoked and outcome-evoked dopamine responses across regions for uncued reward, cued reward, omission, air puff, and omission with air puff. Gray lines connect data from the same animal; black lines show group means ± SEM. ∗: *p* < 0.05 using repeated-measures ANOVA followed by Holm-corrected post-hoc tests to compare response across regions. #: *p* < 0.05 paired *t* tests with Bonferroni correction to compare amplitude of response from baseline. Detailed *p* values are in Tables S2 and S3. *N* = 14 sessions from 7 mice. See also Figures S1–S3.

To record dopamine release simultaneously in multiple brain regions, we expressed GRAB-DA3m^25^ (hereafter referred to as GRAB-DA) and tracked temporal dynamics of dopamine release with multi-site fiber photometry (Figure 1A). While this approach does not permit inference about how individual dopamine neurons or axonal subpopulations encode specific outcomes, it provides a measure of the aggregate, population-level dopamine signal within a defined brain region. With this approach, we targeted the DS, the lateral part of the NAc (NAc_lat_), BLA, and mPFC (Figures 1A and S1A–S1C; Table S1). This selection of targets allowed us to simultaneously sample signals along the nigrostriatal (DS), mesolimbic (NAc_lat_), and mesocorticolimbic pathways (BLA, mPFC) (Figure 1B). All sites were confirmed after recordings by aligning the position of the tip of the optic fiber to coronal sections of the Allen Reference Atlas^37^ using point registration (Figure S1A).

To confirm reliable detection of dopamine release in areas with sparse dopaminergic innervation, such as the mPFC,^38^^,^^39^ we combined optogenetics with fiber photometry. We injected a virus containing the red-shifted activating opsin ChRmine2.0^40^ in the ventral tegmental area (VTA) or the substantia nigra (SN) of DAT-IRES-Cre mice, and implanted an optic fiber above the injection site (Figure S1D). A second fiber measured GRAB-DA responses to photoactivation of dopaminergic neurons in target regions (Figure S1E). A single 10-ms light pulse evoked robust GRAB-DA signals in NAc_lat_ and DS, as expected, and also in the mPFC, demonstrating reliable detection (Figure S1F). Next, we used photoinhibition to assess the ability of our experimental approach to detect dopamine over noradrenaline signals in regions where both neuromodulators are present, such as the mPFC. We used Dbh-Cre mice to express the inhibitory opsin Jaws specifically in noradrenergic neurons of the locus coeruleus and implanted an optic fiber above the injection site (Figure S1G), and a second optic fiber was used to measure GRAB-DA. Mice were water restricted, and we measured the GRAB-DA response to either a water reward or an air puff in the mPFC while silencing noradrenergic neurons (Figure S1H). We found only a limited reduction in the GRAB-DA response to reward or air puff when photoinactivating noradrenergic neurons (Figure S1I). Therefore, our experimental approach allowed us to detect dopamine signals with suitable reliability and specificity.

Head-fixed and water-restricted mice were trained over several sessions on a classical conditioning task in which a 1.5-s pure tone, the conditioned stimulus (CS+), was followed by a water reward (Figure 1C). After several conditioning sessions, mice strongly associated the auditory cue with the reward, as characterized by rapid and sustained anticipatory licking during the cue that was comparable to consummatory licking following reward delivery (Figures S2A and S2B). Following cue-reward association, we modified the task structure to include trials with outcomes varying in sensory intensity and RPE, while recording dopamine signals (Figures 1D–1G and S2C). RPE in our task refers to the expectancy-dependent motivational significance of a stimulus—ranging from strongly positive, such as receiving an unexpected reward, to strongly negative, as when an air puff was delivered in place of an anticipated reward (Figure 1D). Importantly, these outcomes are not assumed to lie on a single psychological value axis; rather, they differ in the degree to which they violate reward expectancy and carry motivational significance. Sensory intensity refers to the physical strength of a stimulus, independent of its reward or aversive value (Figure 1D). For example, water delivery should produce the same sensory intensity regardless of whether it is cued. Direct cross-modal comparison of sensory intensity across modalities (e.g., reward versus air puff delivery) is not straightforward and cannot be established without formal equating procedures. In our framework, we assume that air puff delivery at 50 psi produces a stronger physical stimulus than a small water droplet, based on the relative magnitude of the tactile and auditory impact of each stimulus.

Our analysis of cue- and outcome-evoked dopamine responses showed that dopamine increased significantly following almost all stimulus types across all regions examined (see # in Figure 1H, see also Tables S2 and S3). Cue responses and cued-reward responses were broadly similar across regions, indicating that these signals are encoded in a relatively uniform manner (Figure 1H). Dopamine encoding of uncued reward, although present in all four regions, is reduced in the mPFC (Figure 1H). Reward omission produced the expected decrease in dopamine in the DS and NAc_lat_, but responses in the BLA and mPFC were reversed, showing an increase instead (Figure 1H). Notably, air-puff delivery also evoked significant increases in dopamine in every region, indicating that aversive stimuli were not encoded as negative prediction errors (Figure 1H). In the next section, we will examine these dopamine responses and how they scale with outcome value.

### Dopamine responses partially reflect reward prediction error

**Figure 2:**
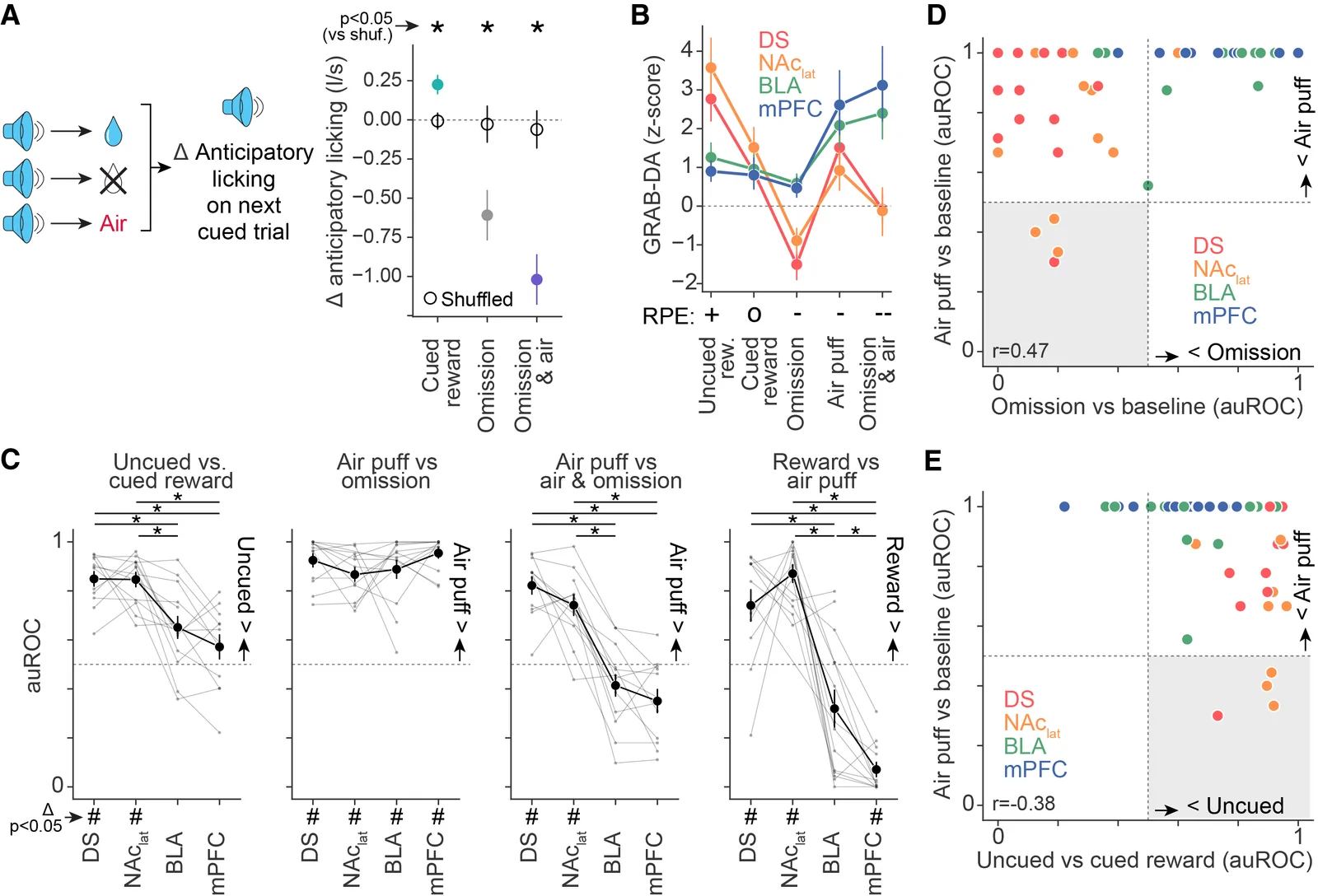
Dopamine dynamics across regions during outcomes does not reflect reward prediction error (A) We calculated the change (Δ) in anticipatory lick rate on the subsequent trial following either cued reward, reward omission, or reward omission combined with an air puff. ∗: *p* < 0.01 using *t* tests with Bonferroni correction to compare change in lick rate following a stimulus versus a shuffled sequence. *N* = 55 sessions. (B) Average *z* scored GRAB-DA responses across trial types varying in RPE for each region (mean ± SEM). (C) auROC comparisons for selected trial outcomes for each region; gray lines indicate individual animals, black lines show group means ± SEM; ∗: *p* < 0.05 using repeated-measures ANOVA followed by Holm-corrected post-hoc tests to compare response across regions. #: *p* < 0.05 paired *t* tests with Bonferroni correction to compare amplitude of response from baseline. Detailed *p* values are in Tables S2 and S3. (D–E) Scatterplots of auROC values for air puff vs. baseline against omission vs. baseline (D) and uncued vs. cued reward (E); each dot represents one session, colored by region. Quadrants highlighted in gray indicate response patterns consistent with reward prediction error encoding. *N* = 14 sessions from 7 mice in (A–E). See also Figures S2 and S3.

Dopamine has been generally linked to reinforcement learning by signaling the difference between expected and actual reward. In this context, it is proposed that dopamine signals should scale with the evaluated significance of an outcome: better-than-expected outcomes evoke larger dopamine responses, expected outcomes evoke smaller ones, and negatively valenced outcomes reduce these responses even further. This RPE should then influence subsequent behavior associated with a context. To test this prediction, we measured how cued reward, reward omission, and the combination of reward omission with an air puff impact subsequent cue-reward association. We found that cued reward slightly increased anticipatory licking rate on the following trials, whereas reward omission and combining omission with an air puff strongly decreased anticipatory lick rate (Figure 2A), and this effect extended for two trials following an air puff (Figure S2D). These findings confirm that the different outcomes of our task carry varying motivational value depending on both expected reward probability and valence.

By examining how dopamine responses varied between our trial outcomes, we found distinct scaling of dopamine signals with RPE across the four regions (Figure 2B). For rewarded outcomes, dopaminergic signals in DS and NAc_lat_ closely followed those expected from classical RPE, with larger responses to uncued than cued reward (Figure 2C; Tables S2 and S3) and dopamine dropping below baseline during reward omission trials (Figure 1H). BLA and mPFC showed no significant scaling of reward responses with expectation (Figure 2C), although uncued reward increased dopamine in these regions (Figure 1H).

These differences in scaling with reward expectation may partly reflect region-specific dopamine kinetics. In particular, the slower kinetics observed in the BLA and mPFC (Figures S2E and S2F) could reduce their sensitivity to decreases in dopamine levels, and trial-wise baseline correction of GRAB-DA signals could mask slow dopamine dynamics. To verify that our baseline correction approach did not introduce such artifacts, we examined raw GRAB-DA signals over extended time windows encompassing entire trials and inter-trial intervals (Figure S3). In all regions, including the mPFC and BLA, dopamine signals reliably returned to baseline prior to the subsequent pre-trial period, and baseline activity showed no systematic drift across the session (Figures S3D and S3E).

We next tested how the delivery of an air puff, either uncued or following the reward-predicting cue, impacts dopamine signals. Because an air puff reduces reward-seeking behavior (Figure 2A), we expected dopamine signals to be decreased, similar to reward omission. Surprisingly, however, an air puff elicited a significant increase in dopamine across all regions, both relative to baseline (Figure 1H) and relative to omission trials (Figure 2C). In DS and NAc_lat_, this response was attenuated when paired with a reward-predicting cue, likely reflecting a combination of omission and air puff signals. Air puff responses were smaller than reward responses in DS and NAc_lat_, but exceeded reward responses in mPFC (Figure 2C). These discrepancies between RPE and dopamine signals were further validated by an absence of correlation between the amplitude of dopamine responses to each outcome and subsequent licking behavior (Figure S2G).

Having established that dopamine does not only reflect RPE, we next more closely examined how dopamine responses to an air puff vary as a function of another negative outcome, reward omission (Figure 2D). Although air puff responses tended to decrease as omission responses became more negative, few recordings showed both signals below baseline, as indicated by the sparse lower-left quadrant (4 out of 50 recording sites across all sessions) (Figure 2D). In addition, dopamine encoding of an air puff was negatively correlated with the scaling of reward with expectation, but few recordings showed both decreased dopamine responses to air-puff delivery and larger dopamine responses to uncued versus cued rewards (Figure 2E). Together, these findings indicate that while striatal dopamine generally tracks RPE in the context of cue-reward association, it fails to produce a negative response for aversive events.

### Dopamine signals across brain regions reflect distinct sensory and reward prediction error components

Recordings of dopaminergic neurons in the VTA and substantia nigra pars compacta (SNc) have shown that the phasic dopamine response can be decomposed into distinct subcomponents.^34^^,^^35^^,^^36^ One component reflects the physical sensory impact of a stimulus, while the other relates to RPE. This two-component framework offers an alternative explanation for dopamine activation by aversive stimuli and other non-rewarding stimuli, as reward and air puff delivery are also sensory stimuli (both auditory and tactile). Dopamine released in the BLA and mPFC could then be explained by an overrepresentation of the sensory component, while dopamine in the DS and NAc_lat_ could more strongly reflect RPE.

**Figure 3:**
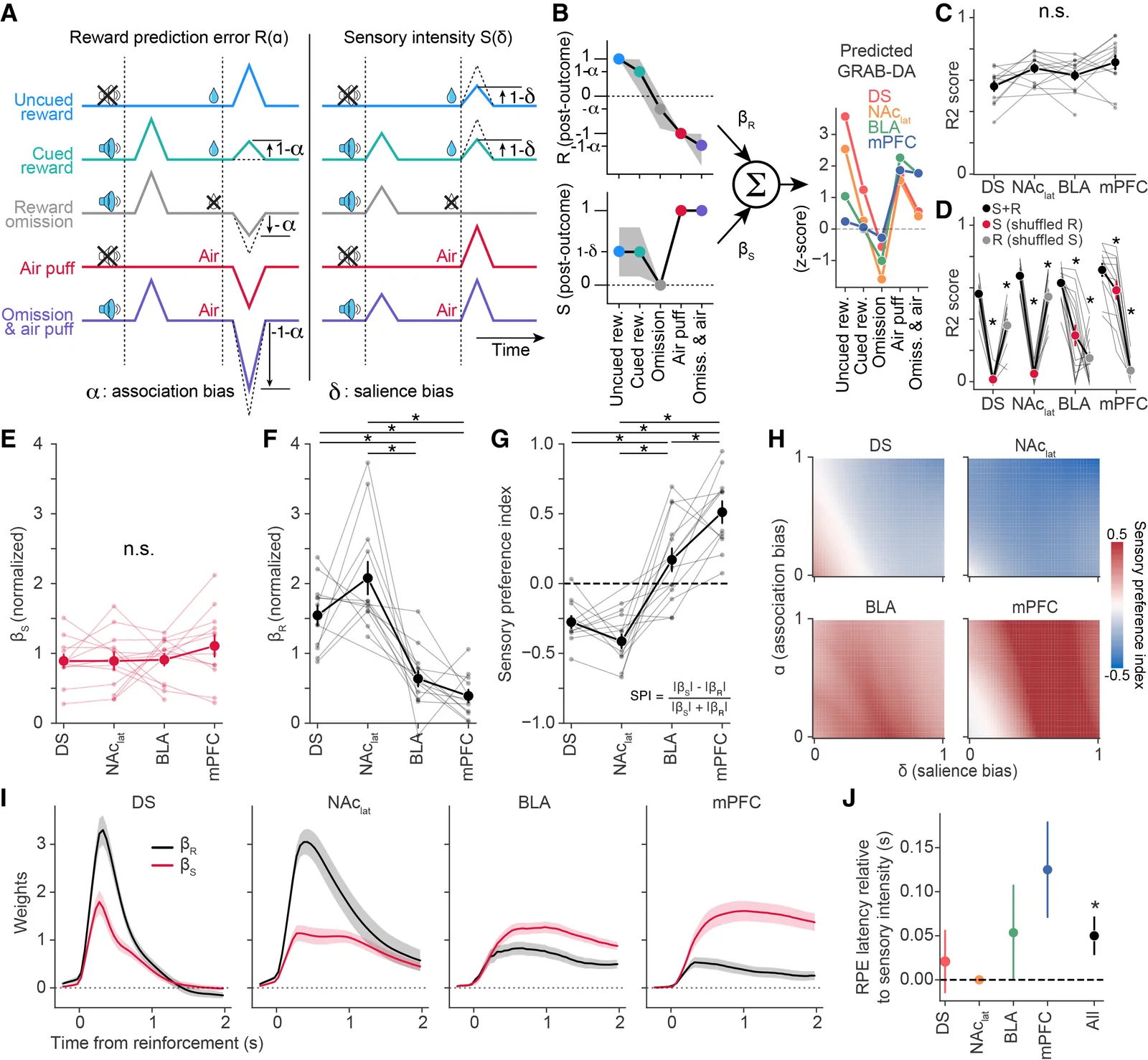
Modeling dopamine responses as a combination of sensory intensity and reward prediction error (A) Schematic of trial types varying in sensory intensity (S) and RPE (R). RPE reflects reward expectation (as a function of associative bias α) and valence, while sensory intensity is assumed constant for each stimulus type (adjusted with a salience bias δ). (B) Dopamine response is expressed as a weighted sum of sensory intensity and RPE components (β_S_ and β_R_). Right image shows predicted GRAB-DA responses across regions for each trial type for one example session. (C) Prediction accuracy (R^2^) of our model across regions. Gray lines indicate individual animals; black lines show group means ± SEM. (D) Average prediction accuracy for modeling GRAB-DA signal using the full model (black), a model with shuffled R (red), and shuffled S (gray). ∗: *p* < 0.05 using a two-sided paired *t* test with Bonferroni correction to compare full versus partial models. (E–G) Estimated parameters across regions: normalized β_R_ (E), normalized β_S_ (F), and sensory preference index (see STAR Methods) (G). Gray lines indicate individual animals; black lines show group means ± SEM; ∗: *p* < 0.05 using repeated-measures ANOVA followed by Holm-corrected post-hoc tests to compare response across regions. (H) Heatmaps showing the averaged sensory preference index across combinations of association bias (α) and salience bias (δ) for each region. (I) Time course of β_R_ (black) and β_S_ (red) weights aligned to reinforcement for each region (mean ± SEM). (J) Latency of RPE component relative to sensory intensity component, computed as the time offset maximizing the cross-correlation between β_R_(t) and β_S_(t) shown in (I). Positive values indicate that the RPE component peaked after the sensory intensity component (mean ± SEM). ∗*p* < 0.05 using a paired *t* tests. *N* = 14 sessions from 7 mice in C–K. See also Figures S4 and S5.

To test whether dopamine signaling can be decomposed into sensory intensity and RPE components, and whether regional differences arise from varying balances between these components, we built a linear model of dopamine signals following reinforcement (Figure 3A). To do this, we defined sensory intensity and RPE as a function of the five different outcomes in our task (Figure 3A). These two functions were further modulated by two bias terms: (1) an association bias (α, bounded between 0 and 1) capturing the strength of cue-reward association used in defining RPE; and (2) a salience bias (δ, also bounded between 0 and 1) to incorporate subjective differences in physical salience between water versus air puff delivery in defining sensory intensity (Figure 3A). We then used constrained least-squares regression to express dopamine responses as a weighted sum of RPE and sensory intensity (Figure 3B). Critically, α and δ were estimated once per recording session using a joint fit across all simultaneously recorded regions, and were then held constant across regions within that session. This approach reflects the assumption that cue-reward association strength and relative physical salience of air-puff versus reward are animal- and session-specific variables, rather than region-specific properties of dopamine signaling. Both association and salience biases were estimated empirically by performing a grid search over α and δ values and selecting the pair that maximized overall model fit across regions (Figure S4). We obtained an average association bias α of 0.82 ± 0.03, indicating a strong cue-reward association. Note that association bias values correlated positively with anticipatory lick rate, linking the modeled association strength to mouse behavior (Figure S4A). The average value for salience bias δ was 0.83 ± 0.03, indicating a higher sensory sensitivity to air puff delivery.

We note that the first component of phasic dopamine responses, here defined as *S(δ)*, can also reflect novelty, surprise, and reward context in addition to physical sensory intensity. However, because animals were extensively exposed to the stimuli during training prior to recordings, novelty- and surprise-driven contributions to dopamine responses were minimized, and contextual variables were held constant across conditions within animals. Under these conditions, physical sensory intensity represents the primary source of variation in the first dopamine component across our trial types, justifying its use as the dominant variable in the sensory intensity regressor.

Our linear model decoded dopamine responses to different outcomes with comparable performance across all four regions (Figures 3C and S4B). Prediction accuracy dropped when either sensory or RPE component was shuffled, indicating that dopamine outcome signaling cannot be explained by a single component (Figure 3D). To examine the balance between sensory and RPE encoding, we extracted normalized regression coefficients (RPE (β_R_) and sensory (β_S_)) (Figures 3E and 3F). On average, β_S_ did not differ significantly across regions (Figure 3E). In contrast, β_R_ was larger in DS and NAc_lat_ compared to BLA and mPFC (Figure 3F). We also assessed how varying association and salience bias values affected the fit quality and regression coefficients (Figures S4C–S4F). Although both β_R_ and β_S_ were influenced by bias values and differed across regions, their linear correlation ensured that our conclusions were unaffected (Figures S4E and S4F). Finally, by computing an index to quantify the balance between sensory and RPE components for each region, we found a preference for sensory intensity encoding in BLA and mPFC (Figure 3G). These larger values for the sensory preference index in cortico-amygdalar circuits were observed for most combinations of values for association and salience bias (Figure 3H).

To further assess the robustness of our findings, we repeated the analysis using an alternative model in which the RPE regressor was set to zero for all punishment conditions, removing any assumption about the negative motivational significance of air puff delivery (Figure S4G). This alternative specification also substantially reduced collinearity between regressors, as the air puff conditions—which received the most extreme values on the RPE regressor in the original model—were the primary driver of regressor correlation. Indeed, the session-by-session Pearson’s correlation between RPE and sensory intensity regressors was reduced from −0.62 ± 0.02 in the original model to −0.30 ± 0.02 in the alternative model (Figure S4H). Critically, despite this substantial change in regressor structure and collinearity, regional differences in sensory preference indices were essentially unchanged, with BLA and mPFC showing higher sensory preference than DS and NAc_lat_ in both models (Figure S4I). Together, these analyses confirm that our conclusions do not depend on how aversive outcomes are assigned on the RPE regressor and are robust across model specifications with varying degrees of regressor orthogonality.

In a different set of experiments, we extended these results to other regions innervated by dopamine axons by recording in the tail striatum (TS), the core of the NAc (NAc_c_), and the olfactory tubercle (OT), and comparing with our results for the DS, NAc_lat_, BLA, and mPFC (Figures S5A and S5B; Table S4). We used the same encoding model (Figures 3A and 3B) to test whether dopamine signaling can be decomposed into sensory intensity and RPE in these additional target regions (Figures S5C and S5D). We found that our linear model reliably explained variance in the signals for these regions (Figures S5E and S5F). Sensory preference indices varied across regions, with the OT showing the lowest values and the mPFC the highest (Figure S5G), suggesting a gradient in the relative weighting of sensory intensity and RPE across the dopaminergic system.

Next, to test whether sensory and RPE components were processed sequentially, we regressed dopamine signals at different time points after outcome delivery (Figure 3I). Given the relatively slow kinetics of GRAB-DA3m sensors (τ_on_: 54 ms; τ_off_: 570 ms measured *in vitro*),^25^ we observed moderate temporal differences between the two components (Figure 3I), unlike reports from spiking data in dopamine neurons.^35^^,^^36^ However, cross-correlation analysis revealed that the sensory component preceded the RPE component by an average of 50 ± 2 ms, supporting the idea that sensory processing occurs before RPE (Figure 3J). Overall, our findings reveal that dopamine sequentially encodes sensory intensity and RPE, with the balance between these components differing across regions: RPE predominating in DS and NAc_lat_, and sensory intensity predominating in BLA and mPFC.

### Dopamine scaling with reward size confirms region-specific reward prediction error encoding

Next, we further assessed whether the RPE component of dopamine signaling was differentially scaled across our target regions. To do this, we manipulated reward value directly while holding sensory properties constant. This approach allowed us to examine RPE encoding independently of the sensory intensity component. The same mice used in the classical conditioning task (Figures 1, 2, and 3) were presented with uncued reward trials in which reward size varied pseudo-randomly (from 0.3 to 10 μL of water).

**Figure 4:**
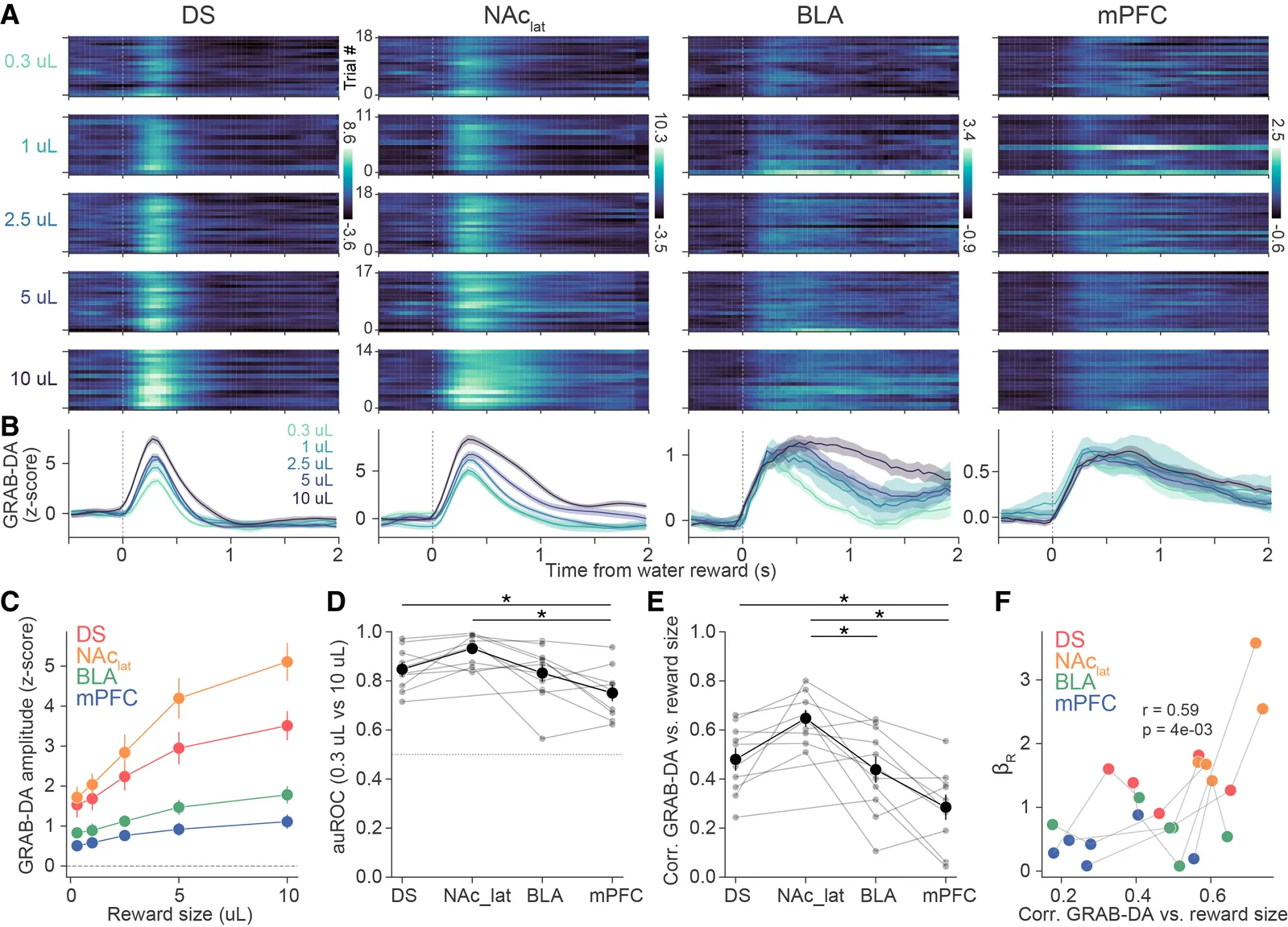
Dopamine scaling with reward size confirms region-specific reward prediction error encoding (A and B) Raster plots (A) and average traces (mean ± SEM) (B) of an example session showing simultaneously recorded GRAB-DA responses aligned to water delivery for five reward sizes (0.3, 1, 2.5, 5, and 10 μL) across DS, NAclat, BLA, and mPFC. Color bar indicates min and max *Z* score values. (C) GRAB-DA response against reward size averaged across all sessions and animals for each region. Data are means ± SEM. (D) auROC comparisons between smallest and largest reward sizes (0.3 vs. 10 μL) for each region; gray lines indicate individual animals, black lines show group means ± SEM. A value above 0.5 indicates preferential encoding of 10 μL reward. (E) Pearson’s correlation coefficient between reward size and GRAB-DA amplitude, computed for each session and region; gray lines connect sessions from the same animal, black points show group means ± SEM. (F) Correlation between the reward size-response correlation shown in (E) and β_R_ (RPE weight, see Figure 3) estimated from the model; each dot represents the average for one region in an animal. Data from the same animal are connected with gray lines; Pearson’s *r* and *p* value shown. ∗: *p* < 0.05 using repeated-measures ANOVA followed by Holm-corrected post-hoc tests to compare response across regions for (D) and (E). *N* = 11 sessions from 6 mice in (C–F).

GRAB-DA recordings revealed that dopamine release scaled monotonically with reward size (Figures 4A–4C). In all recorded regions, dopamine responses were higher for large (10 μL) compared to small (0.3 μL) rewards (*p* = 6.9e−6, 2.6e−5, 1.7e−4, and 4.9e−3 for DS, NAclat, BLA, and mPFC, respectively, using *t* test with Bonferroni correction). These findings suggest that dopamine encodes reward size in all target regions, but with weaker scaling in the mPFC compared to DS and NAc_lat_ (Figure 4D). To quantify how consistently dopamine responses scaled with reward size, we computed, for each session and region, the Pearson’s correlation between reward size and GRAB-DA amplitude (Figure 4E), revealing marked regional differences in the strength of this relationship. This reward size-response correlation was significantly weaker in the mPFC and BLA than in striatal regions (Figure 4E), indicating that dopamine responses in these regions are less consistently graded across reward size.

We next asked whether the degree of reward size scaling predicted variability in RPE encoding across regions (Figures 3F and 4F). We indeed observed a significant correlation between reward size-response correlation and β_R_, providing independent validation that RPE is differentially weighted in striatal versus non-striatal dopamine circuits. Together, these results indicate that while dopamine encodes reward size across all examined pathways, the shape of the reward response function varies systematically across targets—directly corroborating the regional differences in RPE encoding identified by our regression model.

### Dopamine displays high noise correlations within striatal and cortico-amygdalar circuits

Our analyses so far revealed that dopamine responses across regions carry shared sensory information, while RPE is more strongly represented in striatal targets than in non-striatal regions. This raised the question of whether these encoding differences are reflected in the coordination of dopamine fluctuations across regions. Specifically, do striatal and cortico-amygdalar dopaminergic circuits form functionally distinct modules?

To address this, we analyzed trial-to-trial correlations, often referred to as noise correlations.^41^ Noise correlations measure how responses in two signals, in our case dopamine in two distinct regions, co-vary across repeated presentations of the same stimulus, providing insight into functional coupling. Previous studies have reported positive noise correlations between firing rates of dopaminergic neurons during reinforcement learning, but these findings were limited to small neuronal samples.^35^^,^^42^ Here, we leveraged simultaneous GRAB-DA recordings to test whether such correlations exist in dopamine release across distinct brain regions.

**Figure 5:**
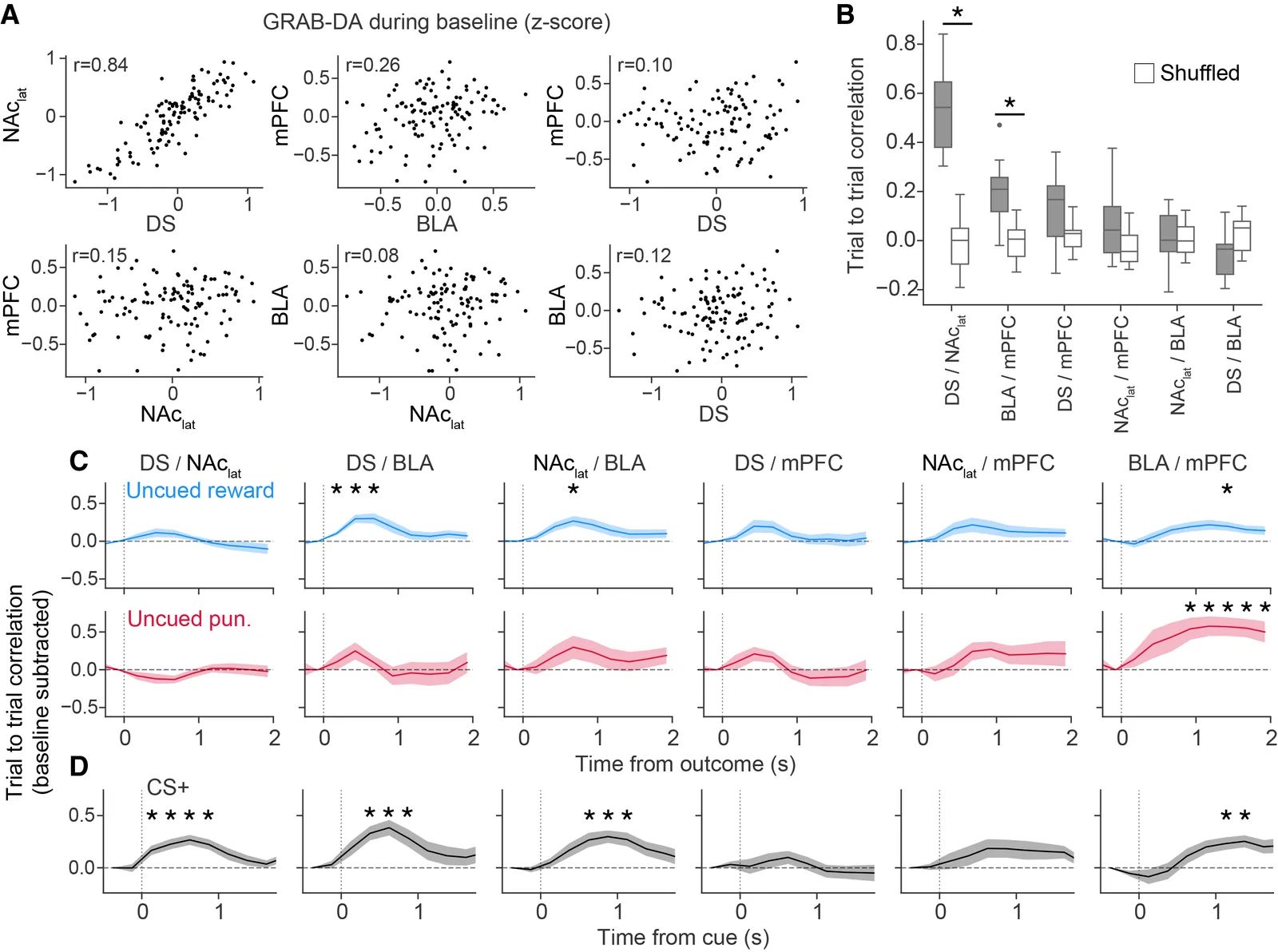
Trial-to-trial correlations of dopamine signals across brain regions (A) Scatterplots showing trial-to-trial correlations between dopamine responses in pairs of regions during a 1-s baseline period. Each dot represents dopamine amplitude for a single trial; Pearson’s correlation coefficient (*r*) is indicated for each pair. (B) Trial-to-trial correlation coefficients across sessions during baseline and for each region pair (gray: actual data; white: shuffled control). Box and whisker plots indicate the median, the 25th and 75th percentile, and the minimum to maximum values of the distribution. ∗: *p* < 0.001 paired *t* test with Bonferroni correction comparing correlation with shuffled trial order. (C and D) Time-resolved noise correlations for all region pairs following uncued reward (top row in C), uncued air puff (bottom row in C), and reward-predicting cue (D). Correlations are computed for each time point and baseline-subtracted relative to the pre-stimulus period. Shaded regions indicate ±SEM across recording pairs. ∗: *p* < 0.05 paired *t* test with Bonferroni correction comparing correlation with baseline at different timing after a stimulus. *N* = 10 DS-NAclat, 10 DS-mPFC, 10 NAclat-mPFC, 12 BLA-mPFC, 12 NAclat-BLA, and 12 DS-BLA recording pairs for (B–D). See also Figure S6.

During a 1-s baseline period preceding any stimulus, we observed significant positive correlations compared to shuffled trial sequences for NAc_lat_-DS and mPFC-BLA pairs (Figures 5A and 5B), with no correlation for other region pairs. These findings suggest that NAc_lat_-DS and mPFC-BLA dopamine fluctuations are selectively coupled. We next examined whether this coupling is modulated by task events varying in sensory intensity and RPE by subtracting the correlation during the baseline period. Increases in baseline-subtracted noise correlation would suggest shared drive to both regions during a given task event. Time-resolved analysis revealed that trial-to-trial correlations increased during reward-predicting cues and uncued reward for several recording pairs, whereas correlations remained stable during cued reward and reward omission trials (Figures 5C, 5D, S6A, and S6B). These findings indicate that dopamine signals across several regions become more synchronized during epochs associated with rewarding outcomes and reward-predicting cues, potentially reflecting a shared value-sensitive drive to dopaminergic neurons projecting to these regions. In contrast, mPFC-BLA recording pairs showed a selective increase in trial-to-trial correlation following air puff stimuli (Figures 5C and S6A). Together, our results support the existence of two coordinated modules of dopamine signaling, NAc_lat_/DS and mPFC/BLA, whose functional organization mirrors the distinct sensory intensity and RPE encoding profiles identified in previous sections. The selective enhancement of mPFC-BLA correlations following an air puff further suggests that cortico-amygdalar dopamine coordination is preferentially driven by salient sensory events, consistent with the higher sensory preference index observed in these regions.

## Discussion

Using multi-site recordings of dopamine release in the DS, NAc_lat_, BLA, and mPFC during outcomes varying in sensory intensities and motivational significance, we provide a systematic characterization of how dopamine dynamics support reinforcement learning and sensory processing, and how these signals are differentially distributed throughout the brain. We found that rather than broadcasting a unitary signal, dopamine conveys a graded mixture of sensory and RPE information whose balance depends on projection target, with striatal regions (DS and NAc_lat_) exhibiting a stronger outcome expectation component than non-striatal regions (BLA and mPFC). Consistent with this, we observed a tendency for stronger RPE encoding in NAc_lat_ compared to DS, suggesting that finer distinctions within the striatum may exist but remain below the resolution of population-level dopamine release measurements, as opposed to axonal imaging approaches that can reveal subregional heterogeneity within the dorsal and ventral striatum.^43^ Together, this organization could allow dopamine to differentially modulate sensory gain in corticolimbic circuits while biasing learning and reinforcement in the striatum.

Variation in the balance between sensory intensity and RPE components of dopamine signaling suggests that dopamine may differentially influence sensory processing and reinforcement learning across target regions. The predominance of RPE signals in striatal regions is consistent with the established role of the striatum in learning which actions or contexts lead to reward.^44^ In turn, dopamine release can reinforce these associations by selectively strengthening corticostriatal synapses that encode action-outcome or stimulus-outcome contingencies.^45^^,^^46^ In contrast, the higher dopamine responses to physically intense sensory stimuli observed in non-striatal regions suggest a role for dopamine in modulating sensory processing. Dopamine has been shown to enhance sensory coding and increase signal-to-noise ratios in the mPFC,^47^^,^^48^ and to reduce inhibitory tone in the BLA.^49^^,^^50^ Accordingly, stronger sensory stimuli may prime these networks for rapid sensory detection, thereby supporting the association of outcome with prominent or novel sensory cues.

Given that phasic dopamine activation is itself reinforcing and promotes reward seeking,^5^^,^^6^^,^^8^ dopamine encoding of RPE in the striatum could support action-outcome and stimulus-outcome learning with relatively weak dependence on sensory intensity. For example, NAc dopamine has been shown to guide learning during probabilistic decision making, where outcomes are inferred from uncertain or weakly informative cues rather than driven by salient sensory stimuli.^51^ By contrast, dopamine in the BLA and mPFC, being more strongly engaged by physically intense sensory events, may tag prominent cues with their identifying features and allocate attention, thereby facilitating subsequent binding of these cues to rewarding^52^ or even negative outcomes in a circuit-specific manner.^33^^,^^53^^,^^54^ Importantly, our results indicate that sensory and RPE components of dopamine signaling are not mutually exclusive. This graded mixing of sensory and outcome information suggests that dopamine does not fully parcel distinct functions to separate targets, but instead broadcasts multiplexed signals whose functional impact depends on local circuit properties.

In addition to directly comparing dopamine responses to reinforcing stimuli across brain regions within the same animals, our multi-site recording approach enabled examination of trial-to-trial correlations in dopamine release. We found that BLA-mPFC and DS-NAc_lat_ pairs exhibited high trial-to-trial correlations during baseline activity, whereas reward-predicting cues and rewarding outcomes selectively increased correlations in several other recording pairs. This pattern is consistent with previous reports showing that cue presentation and reward delivery enhance correlated activity among dopaminergic neurons.^35^^,^^42^ As dopamine neurons primarily projecting to the striatum form dense axonal arbors with little collateralization outside the striatum,^55^^,^^56^^,^^57^^,^^58^ the widespread coupling between DS and NAc_lat_ suggests that shared inputs may drive SNc and VTA neurons projecting to these regions, respectively. Tracing studies indeed suggest that both long-range and local inputs converge onto SNc and VTA dopamine neurons,^18^^,^^59^^,^^60^ potentially accounting for the high correlations observed between projection-specific dopaminergic neurons originating from different midbrain nuclei. By comparison, the coupled dopamine activity observed between the BLA and mPFC may reflect shared cellular origins. Dopamine neurons projecting outside the striatum have more diffuse and collateralized projection patterns, often simultaneously targeting the ventral striatum, amygdala, and prefrontal cortex.^57^^,^^61^ Consistent with this anatomical organization, single-cell gene expression profiling has identified transcriptionally distinct dopamine subtypes shared between BLA- and mPFC-projecting populations.^62^^,^^63^

### Limitations of the study

Several limitations of our modeling approach should be acknowledged. First, the RPE regressor *R*_*i*_*(α)* captures expectancy-dependent modulation of dopamine signals broadly, but cannot disambiguate its psychological subcomponents. In particular, because uncued air puff and cued punishment are physically identical stimuli, any difference in dopamine responses between these conditions is necessarily captured by the RPE regressor. Yet, whether this difference reflects negative RPE, aversive surprise, expectation violation, or broader motivational context cannot be resolved from our data. Second, the sensory intensity and RPE regressors are not fully orthogonal, since the conditions that differ most in motivational context, specifically the aversive conditions, also receive the most extreme values on the RPE regressor. As a result, variance attributable to unmodeled factors such as expectation violation or motivational context could in principle be partially absorbed by the RPE regressor. To assess the robustness of our conclusions to this assumption, we repeated the analysis with the RPE regressor set to zero for all punishment conditions, effectively treating aversive outcomes as carrying no RPE signal. We obtained highly similar regional differences in sensory preference indices under this alternative model (Figure S4I), indicating that our main conclusions do not depend on how aversive outcomes are assigned on the RPE regressor. Third, the sensory intensity regressor captures physical stimulus properties but does not explicitly model other contributors to the first dopamine component such as novelty, surprise, or reward context. While these factors were minimized by extensive behavioral training and a fixed task structure, future work using paradigms that systematically vary novelty or contextual salience will be needed to fully specify this component.

Finally, although fiber photometry offers a powerful tool for estimating average dopamine release within a localized region (∼hundreds of microns), it may underrepresent finer spatial and temporal heterogeneity of dopamine signaling. Dopamine neurons can be molecularly subdivided based on gene expression profiles,^63^ and while these subtypes often project to different targets, their projection fields can also overlap within the same region.^62^^,^^64^^,^^65^ Dopamine signals measured using fiber photometry therefore likely reflect an average across these subtypes, potentially preventing the detection of functional differences between subpopulations. Imaging of individual dopamine axons in the DS and mPFC has shown marked diversity in how different axons encode rewarding and aversive signals.^28^^,^^64^^,^^66^ Moreover, imaging of dopamine release in the DS using two-photon microscopy has revealed wave-like dynamics in response to reward.^67^ Dopamine acts via both synaptic and volume transmission,^68^ raising questions about how local fluctuations in dopamine levels relate to neuromodulatory signaling at the cellular level. Future work incorporating higher spatial and temporal resolution techniques with genetic dissection of dopaminergic circuits, while maintaining a multi-region approach, will be essential to further resolve the regional weighting of sensory intensity and RPE components across dopaminergic targets.

## Resource availability

### Lead contact

Requests for further information and resources should be directed to and will be fulfilled by the lead contact, Vincent Breton-Provencher (vincent.breton-provencher@cervo.ulaval.ca).

### Materials availability

This study did not generate new unique reagents.

### Data and code availability

• Trial-aligned fiber photometry data have been deposited at The Open Science Framework (OSF) and are publicly available as of the date of publication at https://doi.org/10.17605/OSF.IO/DV724.
• All original code has been deposited at https://doi.org/10.5281/zenodo.21498194 and is publicly available as of the date of publication.
• Any additional information required to reanalyze the data reported in this paper is available from the lead contact upon request.

## Acknowledgments

We thank Christian Éthier, Kyle Jenks, and Gabi Drummond for feedback on the manuscript. Dr. Yulong Li (Peking University) for providing early access to the GRAB-DA3m sensor. Support to the VBP lab was provided by 10.13039/501100000038NSERC Discovery grant no. (RGPIN-2021-03284), 10.13039/501100000024CIHR project grant no. (#517536), CFI John R. Evans Leaders Fund (grant no. 44014), AFOSR Cognitive & Computational Neuroscience Program (FA9550-23-1-0533), a Future Leaders in Canadian Brain Research Program from Brain Canada, and a Research Scholars—Junior 1 Salary Award from Fonds de recherche du Québec (10.13039/501100020951FRQ), Santé. Support to the ML lab was provided by the 10.13039/501100000038NSERC Discovery grant no. (RGPIN-2024-05363), 10.13039/501100000024CIHR project grant nos. (#451548 and #551059) and Research Scholars—Senior Salary Award from Fonds de recherche du Québec (10.13039/501100020951FRQ), Santé and Parkinson Quebec.

## Author contributions

Conceptualization, formal analysis, writing – original draft: S.J.B. and V.B.P.; methodology and investigation: S.J.B., J.B., and V.B.P.; resources, supervision, funding acquisition, writing – review and editing: M.L. and V.B.P.

## Declaration of interests

All affiliations are listed on the title page of the manuscript. All funding sources for this study are listed in the “acknowledgments” section of the manuscript. We, the authors and our immediate family members, have no positions to declare and are not members of the journal’s advisory board. We, the authors and our immediate family members, have no related patent applications or registrations to declare. Martin Lévesque is a co-founder, shareholder, and member of the scientific advisory board of CEREBRO Therapeutics. This research was conducted independently and did not involve CEREBRO Therapeutics.

## Declaration of generative AI and AI-assisted technologies in the writing process

During the preparation of this work, the authors used Claude AI in order to proofread the manuscript. After using this tool or service, the authors reviewed and edited the content as needed and take full responsibility for the content of the publication.

## STAR★Methods

### Key resources table

**Table:** 

REAGENT or RESOURCE	SOURCE	IDENTIFIER
**Antibodies**		

Rabbit anti-GFP	Synaptic System	Cat# 132002; RRID:AB_887725
Goat anti-Rabbit Secondary Antibody, Alexa Fluor 568	Invitrogen	Cat# A-11011; RRID:AB_143157

**Bacterial and virus strains**		

AAV2/9-hSyn-GRAB-DA3m	BrainVTA	Cat# PT4720
AAV2/8-nEF-Con/Foff 2.0-ChRmine-oScarlet	Addgene	Cat# 137161-AAV8
AAV2/5-CAG-Flex-rc[Jaws]-KGC-GFP-ER2	Addgene	Cat# 84445-AAV5

**Chemicals, peptides, and recombinant proteins**		

Phosphate buffered saline (PBS)	Sigma-Aldrich	Cat# P4417
Paraformaldehyde 4% (PFA)	Electron Microscopy Sciences	Cat# 15710
Prolong Gold Antifade Mountant with DAPI	ThermoFisher	Cat# P36931
C&B Metabond adhesive cement	Parkell	Cat# S380

**Deposited data**		

GRAB-DA Fiber Photometry Dataset	OSF	https://doi.org/10.17605/OSF.IO/DV724

**Experimental models: Organisms/strains**		

C57Bl/6J wild-type mice	The Jackson Laboratory	JAX# 000664
Dat-IRES-Cre mice (B6.SJL-Slc6a3tm1.1(cre)Bkmn/J)	The Jackson Laboratory	JAX# 006660
Dbh-Cre KI mice (B6.Cg-Dbhtm3.2(cre)Pjen/J)	The Jackson Laboratory	JAX# 033951

**Software and algorithms**		

Python (versions 3.9–3.12)	Python Software Foundation	https://www.python.org
ImageJ	NIH	https://imagej.nih.gov/ij/
Custom code for analysis	Zenodo	https://doi.org/10.5281/zenodo.21498194

**Other**		

Fiber optic cannulas (0.39 NA, 400 μm)	RWD	R-FOC-BL400C-39NA
Multi-fiber optic patch cord, 4-ended (BBP(4)_400/440/900–0.37)	Doric Lenses	BBP(4)_400/440/900–0.37_2m_FCM-4xMF1.25_LAF
Patch cord for optogenetics (400 μm, 0.39 NA)	Thorlabs	Cat# M98L01
Patch cord for LC silencing (200/220 μm, 0.37 NA)	Doric Lenses	MFP_200/220/900–0.37_2m_FC-ZF1.25
Arduino Nano Every microcontroller	Arduino	Nano Every
Arduino Uno microcontroller	Arduino	Uno
Picospritzer III	Parker Hannifin	N/A
Nanoliter injector	WPI Inc.	NANOLITER2020
Vibratome	Leica	VT1000S
Epifluorescence microscope	Zeiss	Axio Imager M2

### Experimental model and study participant details

#### Animals

All animal procedures were approved by Université Laval’s Animal Protection Committee and conformed with the guidelines established by the Canadian Council on Animal Care. Fifteen male and five female mice were used for recording GRAB-DA. An additional ten male and twelve female mice were used for behavioral analyses (Figures 2A and S2D). Injection sites, atlas coordinates, age, and sex are listed in Tables S1 and S4. Mice were housed in a room with light on from 7 am to 7 pm in ventilated cages with controlled humidity (50 to 70%) and temperature (22 to 25 °C). Food and water were provided *ad libitum* unless noted (see behavioral training and task). All experiments were performed during the light part of the cycle. Most experiments were carried out in C57BL/6 wild-type mice. The *Dat-IRES-Cre* line (B6.SJL-Slc6a3tm1.1(cre)Bkmn/J; JAX stock #006660) was used for specific expression of the activating opsin ChRmine2.0 in dopamine-expressing neurons of the substantia nigra or ventral tegmental area (Figures S1D–S1F). The *Dbh-Cre KI* line (B6.Cg-Dbhtm3.2(cre)Pjen/J; JAX stock #033951) was used for specific expression of the silencing opsin Jaws in noradrenaline-expressing neurons of the locus coeruleus (Figures S1G–S1I).

### Method details

#### Surgeries

Mice were anesthetized with isoflurane (4% induction, maintained with 1.5-2%). General analgesia (carprofen, 2 mg/ml) and local anesthesia (lidocaine, 0.8 mg/ml; bupivacaine 0.4 mg/ml) were given before the beginning of the surgery. Lactated Ringer’s solution was injected at the beginning of the surgery and once per hour during surgery. Following deep anesthesia, mice were placed in a stereotaxic frame (Model 942, Kopf), their heads were shaved and cleaned, and their scalps were removed with surgical scissors. The skull was aligned in the stereotaxic frame by leveling the heights of the bregma and lambda.

Four holes of 0.5 mm diameter were drilled on the skull. Two holes were made on the left hemisphere for NAc_lat and BLA implantation and two on the right hemisphere for mPFC and DS. A virus expressing GRAB-DA3m^25^ (AAV2/9-hSyn-GRAB-DA3m, titer of 5.51 × 10^12^ GC/mL, BrainVTA #PT4720) was injected in the following targets: DS [antero-posterior (AP): 0.2-0.8 mm, medio-lateral (ML): 1.7-2 mm, dorso-ventral (DV): -2.5 mm], NAc_lat_ [AP: 1.57, ML: 1.65, DV: -4.4], BLA [AP: -1.6, ML: 3.35, DV: -4.95], and the prelimbic division of the mPFC [AP: 2.1, ML: 0.3, DV: -1.2]. 200 nL of the virus was injected at a volume rate of 100 nL/min using a nanoliter injector (NANOLITER2020, WPI inc) for each injection site. In the mice used for recording in the TS, NAc_c_, or OT, a hole was drilled in each hemisphere to combine TS recordings with either NAc_c_ or OT recordings. We used the following coordinates: TS [AP: -1.8, ML: 3.25, DV: -2.5], NAc_c_ [AP: 1.57, ML: 1.15, DV: -4.2], and OT [AP: 2, ML: 1, DV: -4.2]. During the same surgery, fiber optic cannulas (0.39 NA, 400 μm, RWD) were implanted 0.2 mm above each injection site for fiber photometry measurements.

For optogenetics experiments in which we activated dopamine-expressing neurons, we injected 400 nL of a cre-dependent virus coding for the opsin ChRmine 2.0 (AAV2/8-nEF-Con/Foff 2.0-ChRmine-oScarlet, titer of 2.1 × 10^13^ GC/mL, #137161-AAV8 Addgene) in the VTA [AP: -3.3, ML: 0.4, DV: -4.2] or the SNc [AP: -3.5, ML: 1.2, DV: -4] of *Dat-Cre* mice. For optogenetics experiments in which we silenced noradrenaline-expressing neurons, we injected 400 nL of a cre-dependent virus coding for the opsin Jaws (AAV2/5-CAG-Flex-rc Jaws-KGC-GFP-ER2, titer of 1.3 × 10^13^ GC/mL, #84445-AAV5 Addgene) in the locus coeruleus [AP: -3.3, ML: 0.4, DV: -4.2] of *Dbh-Cre* mice. A fiber optic cannula (0.39 NA, 400 μm, RWD) was also implanted 0.2 mm above the opsin injection site.

For all surgeries, a custom head plate, which was used for head fixation, was positioned on the skull of the mouse. The cannulas and the head plate were attached to the skull with adhesive cement (C&B Metabond, Parkell). Animals were kept under general analgesia for 48h following surgery. Mice were single housed after surgery for improved recovery and for handling water regulation (see behavioral training and task).

#### Behavioral apparatus

Mice were head-fixed on a behavioral rig and confined in a plastic tube (a modified 50-mL centrifuge tube) to restrict body movements. The behavioral rig was adapted from a previously published setup.^69^ Briefly, a metallic lick spout, placed ∼2 mm away from the mouth and connected to a custom-made lick detector, was used to deliver water rewards. Rewards of various sizes (0.3 μL to 10 μL) were delivered from a 3 mL water reservoir positioned above the behavioral rig and connected to a solenoid valve (MB2029-036B-B-Y1-203, GEMs sensors). Durations of valve opening were calibrated and tested routinely to provide a precise and reliable delivery of reward volumes. A small tube at 3 cm from the mouse facial area was used to deliver air puff (compressed air at 50 psi for 0.3 s). The tube was connected to a Picospritzer III (Parker Hannifin) for controlling the timing and repeatability of air puff pulses. A microcontroller board (Arduino Nano Every) was used to control the timing of reward/air puff delivery, to record voltage signals from the lick detector, and to send a TTL signal at the end of each trial for syncing with the fiber photometry system. Sound stimuli (1.5 s duration, 4 or 12 kHz) were delivered via two speakers positioned 25 cm from each side of the mouse. The sound stimulus intensities were established by a sound level meter. The Arduino microcontroller controlling and recording the behavioral setup was connected to a computer running a custom-written Python (version 3.11 or 3.12) script that was able to record lick rate, while controlling the timing of the sound cue (using the Simple Audio library), as well as the water and air puff delivery. Behavior rigs were assembled primarily with optomechanical components (Thorlabs) and 3D printed parts. Behavior was run inside a foam-lined sound attenuating cubicle (ENV-022, Med Associates).

#### Behavioral training and task

At > 1 week following surgery, mice were water restricted by progressively reducing their daily water intake (from 2.5 mL to 0.8-1.5 mL). During water restriction, the weight of each mouse was maintained above 80% of its initial weight, and body condition score was monitored closely. Mice were habituated to head fixation and the behavior rig for 5-10 minutes during 2 consecutive days before the start of training. During this habituation period, a few water rewards were delivered from the lick spout to initiate licking and to reduce stress.

Each trial started with an inter-trial interval randomly drawn from an exponential distribution (mean: 10 s, range: 0 to 45 s). This was followed by an auditory pure tone of 1.5 s duration. A reinforcement - a drop of water (5 μL for the classical conditioning task or 0.3-10 μL for the variable reward task), an air puff, or nothing - was delivered at the offset of the tone. The end of each trial was followed by a fixed delay period of 4 s. We used a pseudo-randomized trial sequence for all behavioral training and testing. We ensure with this pseudo-randomized order that the proportion of each trial type presented was similar for every block of ten trials.

During training, we used two types of trials: conditioning trials where a 12 kHz auditory cue (the conditioned stimulus, CS+) was immediately followed by a 5 μL water reward and trials where only a 4 kHz tone was presented (neutral cue, CS-). Training sessions consisted of an average of 80 trials per day and lasted until the animal was fully conditioned on the 12 kHz auditory tone. Conditioning was evaluated by comparing the rate of anticipatory licking during the CS+ and consummatory licking following water delivery. A mouse was considered trained if these two rates were nearly equal (Figure 2C). Training lasted for a minimum of 10 sessions and until the mouse was fully conditioned. We used a tone intensity of 35 dB for each cue, calculated by measuring the sound pressure level for either CS+ or CS- cue and subtracting the noise level of that given frequency.

Following training, we recorded the dynamics of dopamine release in mice during two versions of a classical conditioning task. In a first version of the task, we used only cued (CS+) and uncued (no CS) rewarded trials. Trials were equally divided into either cued or uncued reward delivery and the trial sequence was randomized. All trials were rewarded, but with reward volume randomly chosen between 0.3, 1, 2.5, 5 or 10 μL. Note that the data for cued reward was not analyzed for this study. In a consecutive second version of the task, we used the following trials: CS+ followed by either reward, no reward (omission), or by delivering an air puff to the facial area of the mouse; and we also had uncued reward and air puff trials. The sequence of trial types was randomized using these probabilities: 0.35 of uncued reward, 0.35 of cued reward, 0.1 CS+ with omitted reward, 0.1 CS+ with air puff, 0.1 uncued air puff. The average probabilities for all session were: uncued reward 0.320 ± 0.002, cued reward 0.362 ± 0.001, omission 0.131 ± 0.002, uncued air puff 0.072 ± 0.003, and omission with air puff 0.109 ± 0.002. We used a lower proportion of air puff trials to maintain engagement of the animal in the task. Fiber photometry recordings were done every two sessions to avoid bleaching of the GRAB sensors. Mice were recorded for two sessions each on the variable reward task and the reward/air puff classical conditioning task. During non-recorded sessions, we only presented cued reward trials to maintain the association of reward with CS+. It should be noted that animals were also tested on a variation of the classical conditioning task that included CS- trials but excluded air puff trials. These data were not analyzed for this study.

#### Multi-site fiber photometry

Fiber photometry recordings were done at least 4 weeks post-surgery. We used a custom-made fiber photometry system adapted from a previously published design.^29^^,^^70^ Briefly, the light from two mounted LEDs of 415 and 470 nm wavelengths (#M415L4 and #M470L5, Thorlabs) was focused with aspherical lenses (#LA1951 – N-BK7, Thorlabs), bandpass filtered (#FB410-10 and #FB470-10, Thorlabs), and combined with a dichroic mirror (#DMLP425R, Thorlabs). The emission light was then reflected using a dichroic mirror (#FF495-Di03-25x36, Semrock) onto the back aperture of a 10X objective (UPlanXApo 0.40NA/3.1WD) used for collimating the light onto the single optical connector side of a multi-fiber optic patch cord. We used four-ended (BBP(4)_400/440/900-0.37_2m_FCM-4xMF1.25_LAF, Doric) low-autofluorescence patch cords to simultaneously record from four targets. Emission light was collected back onto the microscope objective, bandpass filtered (#FF01-535/22-25, Semrock), and detected with a CMOS camera (Blackfly S BFS-U3-04S2M, Teledyne). To isolate the isosbestic from the GRAB-DA signal, the 415 and 470 nm LEDs were alternated at a rate of 40 Hz for a final acquisition rate of 20 Hz. We set the power at the output of the patch cord to 35-50 μW for the 470 nm light. Once the patch cable was connected to the optic fiber cannula on the animal, the power of the 415 nm LED was adjusted to detect an emission power equivalent to the 470 nm signal. Synchronization of the camera frame rate and the two LEDs was done by a microcontroller (Arduino Nano Every). A second microcontroller (Arduino Uno) was used to synchronize the fiber photometry system with behavior and to interface the system with a computer for data acquisition. A custom Bonsai script was used for saving raw fluorescence signals from individual optic fibers imaged onto the camera as well as the TTL signal from the behavior.

Raw fiber photometry data were processed with a custom-made Python script. The isosbestic and GRAB-DA signals were separated, and each signal was high-pass filtered at 0.005 Hz using a 2^nd^ order Butterworth filter to remove slow drifts in fluorescence intensity. To correct for motion or light artifacts, the isosbestic signal was subtracted from the GRAB-DA signal. The signal was then smoothed with a 5-point moving average. Using the onset of the TTL signal, the corrected GRAB-DA signal was then aligned to the beginning of each trial. The baseline period for each trial was defined by a 0.5-second-long window preceding the tone onset, or reinforcement when no tone was presented. GRAB-DA signal was z-scored by dividing by the standard deviation of the baseline signal. The GRAB-DA signal for each trial was baseline subtracted, except for Figure S3. Note that we included the data for which at least three of the four recording sites provided reliable photometry signals for multi-site analysis (see Table S1 for more details).

#### Optogenetics

To activate dopamine expressing neurons and record from either the NAc_lat_, DS, or the mPFC, we used an LED light source of 590 nm (M590F3, Thorlabs) wavelength controlled by a microcontroller (Arduino UNO). We used a power of 3.15 mW, measured at the tip of the patch cord (400 μm 0.39 NA, M98L01, Thorlabs). The patch cord was connected to the optic fiber cannula positioned above the VTA (for NAc or mPFC experiments) or the substantia nigra (DS) of a head-fixed mouse. Trains of 10 ms light pulses at a frequency of 10 Hz were presented every 15 seconds. We used 1 (0.1 s), 2 (0.2), 5 (0.5), 10 (1) or 20 (2) light pulses, and each pulse train was repeated 30 times. For analyzing the GRAB-DA response in experiments in which we photoactivated DA-expressing neurons, we baseline-subtracted each trial and calculated the area under the curve for a period of 1 s following a single 10-ms pulse of light in the VTA or SNc.

For silencing noradrenergic activity from the locus coeruleus and measuring GRAB-DA signals in the mPFC in response to water or air puff delivery, we used a laser diode light source of 638 nm wavelength (LDFLS_473/070_638/120, Doric Lenses). We used a power of 15 mW, measured at the tip of the patch cord (MFP_200/220/900-0.37_2m_FC-ZF1.25, Doric Lenses). The patch cord was connected to the optic fiber cannula positioned above the LC of a head-fixed mice. Trial sequence alternated between reward, air puff, light on only, light on with reward, and light on with air puff. Each trial type was repeated 15 times using a 20 second inter-trial interval. We used a continuous light pulse that was started 1 second before reward or air puff delivery, and lasted for 3.5 seconds, including a 0.5 second ramp down of the light power. For analyzing the GRAB-DA response in the mPFC when we photoinactivated noradrenergic neurons of the locus coeruleus, we baseline-subtracted each trial using a period preceding the air puff or water delivery. We then calculated the area under the curve for a period of 1 s following the air puff or water delivery and compared laser on versus off trials.

#### Histology

Mice were anesthetized with ketamine/xylazine (10 mg/ml; 1 mg/ml), perfused with standard phosphate buffer solution (PBS, P4417, Sigma-Aldrich), and then perfused with a 4% paraformaldehyde solution (PFA, #15710, Electron Microscopy Sciences). Following perfusion, brains were immersed in 4% PFA for 24 h for post-fixation and then kept at 4 °C in PBS. Fixed brains were sliced coronally at a thickness of 100 μm using a vibratome (Leica VT1000S). For some of the slices, we confirmed the expression of GRAB-DA by staining for GFP with immunohistochemistry. These slices were blocked and permeated at room temperature in a 10% NGS, 0.5% triton solution for 1 h, then incubated with the primary antibody anti-GFP (1:500, rabbit, Synaptic systems, 132 002) at 4°C for 12-24 h. The next day, the slices were washed with a 1% NGS, 0.05% triton solution and were incubated with the secondary antibody AlexaFluor 568 anti-rabbit (1:400, Invitrogen, A11011) for 2 h at room temperature. Slices were mounted using Prolong Gold Antifade Mountant with DNA stain DAPI (P36931, ThermoFisher) and imaged on an Axio Imager M2 epifluorescence microscope (Zeiss) or on a confocal microscope (LSM700, Zeiss). All images were processed with the open-source software ImageJ^71^ to adjust brightness and pixel resolution. Anatomical position of the fiber tip was registered to the Allen Mouse Brain Common Coordinate Framework (CCFv3)^37^ using a custom-made Python script. This script enabled us to browse the atlas in the coronal axis and tilt the coronal plane angle to find the best section of the atlas that matched our brain slice. Then the slice was registered onto this atlas section using point registration.

### Quantification and statistical analysis

All data analysis, unless noted, was performed using custom-made scripts written in Python (version 3.9 to 3.12).

To quantify GRAB-DA responses, we calculated the area under the curve following stimulus onset. A 1-second window was used for auditory cue, cued reward, omitted reward, uncued reward, and air puff responses. Decoding performance was assessed using receiver operating characteristic (ROC) curves, calculated by comparing GRAB-DA amplitude distributions across trials. The area under the ROC curve (auROC) was used to indicate decoding performance, with auROC values close to 0.5 representing chance-level performance.

To quantify the contribution of sensory intensity and RPE encoding to dopamine signals across brain regions, we modeled the observed signal for each region *r* and for each trial outcome *i* using the following:

$${\text{signal}}_{ir}=\beta_{0r}+\beta_{Rr}R_{i}\left(\alpha \right)+\beta_{Sr}S_{i}\left(\delta \right)$$

Where *β*_0*r*_ is the intercept for region *r*, *β*_*Rr*_ and *β*_*Sr*_ are region-specific coefficients for RPE and sensory predictors, and *R*_*i*_(*α*) and *S*_*i*_(*δ*) are trial-specific functions of association (*α*∈[0,1]) and salience (*δ*∈[0,1]) bias. RPE *R*_*i*_(*α*) and sensory intensity *S*_*i*_(*δ*) was defined as:

$$R_{i}\left(\alpha \right)=\left\{ \begin{array}{cc} 1 & {\text{if}\;\text{CS}}^{+}\text{absent}\;\text{and}\;\text{reward}\;\text{present}\;\left(\text{uncued}\;\text{reward}\right),\\ 1-\alpha & {\text{if}\;\text{CS}}^{+}\;\text{and}\;\text{reward}\;\text{present}\;\left(\text{cued}\;\text{reward}\right),\\ -\alpha & {\text{if}\;\text{CS}}^{+}\;\text{present}\;\text{and}\;\text{reward}\;\text{absent}\;\left(\text{reward}\;\text{omission}\right),\\ -1 & {\text{if}\;\text{CS}}^{+}\;\text{absent}\;\text{and}\;\text{air}\;\text{puff}\;\text{present}\;\left(\text{uncued}\;\text{air}\;\text{puff}\right),\\ -1-\alpha & {\text{if}\;\text{CS}}^{+}\;\text{present}\;\text{and}\;\text{air}\;\text{puff}\;\text{present}\;\left(\text{omission}+\text{air}\;\text{puff}\right). \end{array}\right.$$

$$S_{i}\left(\delta \right)=\left\{ \begin{array}{cc} 1 & \text{if}\;\text{air}\;\text{puff}\;\text{present}\;\left(\text{uncued}\;\text{air}\;\text{puff}\;\text{or}\;\text{omission}+\text{air}\;\text{puff}\right),\\ 1-\delta & \text{if}\;\text{reward}\;\text{present}\;\left(\text{uncued}\;\text{or}\;\text{cued}\;\text{reward}\right),\\ 0 & {\text{if}\;\text{CS}}^{+}\;\text{present}\;\text{and}\;\text{reward}\;\text{absent}\;\left(\text{reward}\;\text{omission}\right), \end{array}\right.$$

Because dopamine responses measured with GRAB-DA were asymmetric, we applied a leaky ReLU transformation to the output of the RPE function, using a negative slope scaling factor of 0.5. This factor was derived by computing the ratio of the mean absolute value of negative responses to the mean of positive responses of the z-scored distribution of the GRAB-DA signal. After calculating the reward (*R*) and sensory (*S*) values for each trial within a session, these values were z-scored prior to fitting dopamine signals in each region using least-squares linear regression.

To assess the robustness of our findings regarding the motivational significance of aversive outcomes, we fitted an alternative model in which the RPE regressor was set to zero for uncued air puff trials and to −α for cued air puff trials (Figure S4G). This alternative specification removes the assumption that aversive outcomes carry negative RPE commensurable with appetitive outcomes, while preserving the expectancy-dependent modulation for cued punishment, reflecting the violation of reward expectancy when an air puff is delivered following a reward-predicting cue. All other model parameters and fitting procedures were identical to those described above.

Parameters *α* and *δ* were optimized empirically by testing all combinations in increments of 0.025. Model fit quality was assessed using the *R*^2^ score for each combination. For each recording session, α and δ were optimized using a joint fit across all simultaneously recorded regions, and the resulting values were then held constant across regions within that session. Results for all parameter combinations are shown in Figure S4, from which the optimal association bias *α* and salience bias *δ* were extracted and held constant across regions recorded within the same session to generate the data presented in Figure 3. This approach reflects the assumption that cue–reward association strength (*α*) and relative physical salience of air puff versus reward (*δ*) are animal- and session-specific variables, rather than region-specific properties of dopamine signaling. For recordings done in NAcc, TS, and OT, we used fixed *α* and *δ* values of 0.82 and 0.83 for all recordings. Finally, the sensory preference index, a metric quantifying the relative contribution of sensory intensity to dopamine encoding, was computed using the following equation:

$$Sensory\;preference\;index=\frac{\left|\beta_{S}\right|-\left|\beta_{R}\right|}{\left|\beta_{S}\right|+\left|\beta_{R}\right|}$$

Although *α* and *δ* were optimized using the full dataset for the results reported in Figure 3, we verified that this approach does not introduce circularity by performing 5-fold cross-validation stratified by trial type, in which *α* and *δ* were estimated exclusively on training folds and model R^2^ was evaluated on held-out test folds. Cross-validated R^2^ remained well above zero across all regions and sessions (Figure S4B), confirming that the reported model fits reflect genuine explanatory power rather than overfitting to the data used for parameter estimation.

To assess the temporal relationship between sensory intensity and RPE components, we performed time-resolved regression by fitting the model at each time point within a window of 0 to 1.5 seconds following reinforcement, yielding time courses of βR and βS for each region (Figure 3I). To quantify the temporal lag between the two components, we computed the cross-correlation between *β*_*R*_(t) and *β*_*S*_(t) for each session and region. The lag was defined as the time offset at which the cross-correlation was maximized, expressed as the difference in peak timing between the RPE and sensory intensity components (RPE peak time minus sensory intensity peak time). A positive value therefore indicates that the RPE component peaked after the sensory intensity component.

For each session and region, we computed the Pearson correlation coefficient between reward size (0.3–10 μL) and the corresponding GRAB-DA response amplitude across the five reward sizes tested.

To measure noise correlation, we used data from simultaneously recorded animals in the DS, NAc_lat_, BLA, and mPFC. Trial-by-trial correlation in GRAB-DA response amplitudes was calculated using a 1-second average preceding cue, air puff, or water delivery for baseline. Significance was evaluated by comparing with the correlation obtained from shuffling trial sequences from one of the two recording locations. For time-resolved correlation analyses, signal was binned in 0.5 second increments and baseline subtracted.

#### Statistics and reproducibility

Statistical details for all experiments, including exact sample sizes, statistical tests, and *p*-values, are reported in the figure legends, in the main text, and in Tables S2 and S3.

The sample size refers to the number of recording sessions, animals, or region pairs depending on the analysis, as specified in each figure legend. For multi-site fiber photometry experiments, the primary dataset consisted of 14 recording sessions from 7 mice, and noise correlation analyses included between 10 and 12 recording pairs per region pair. For behavioral analyses, 55 sessions from 22 mice (10 male, 12 female) were used. For optogenetic silencing experiments, *n* = 5 mice were used. Data are presented as mean ± SEM throughout, unless otherwise stated.

Group differences across brain regions were assessed using repeated-measures ANOVA followed by Holm-corrected post-hoc pairwise t-tests. Comparisons of GRAB-DA response amplitudes against baseline used two-sided paired t-tests with Bonferroni correction for multiple comparisons. Noise correlations were assessed by comparing observed trial-to-trial correlations (Pearson's *r*) against a shuffled control distribution using paired t-tests with Bonferroni correction. Comparisons between full and partial regression models used two-sided paired t-tests with Bonferroni correction. For the optogenetic validation experiment, a Wilcoxon signed-rank test was used. Significance was set at *p* < 0.05 for all tests. Complete pairwise statistical comparisons across regions are reported in Table S2, and within-region comparisons across conditions are reported in Table S3.

No formal *a priori* power analysis was conducted to determine sample sizes; sample sizes were chosen based on prior fiber photometry studies in similar experimental paradigms. No animals or recording sessions were excluded, except that sessions were required to have at least three of four recording sites providing reliable photometry signals to be included in multi-site analyses (see Table S1 for details). Trial sequences were pseudo-randomized to ensure balanced trial-type proportions across blocks of ten trials. Animals were not randomly assigned to experimental groups, as all mice underwent the same behavioral and recording protocol. All surgeries and recordings were performed without blinding to experimental condition.

### Additional resources

Trial aligned fiber photometry data have been deposited at The Open Science Framework (OSF) and are publicly available at https://doi.org/10.17605/OSF.IO/DV724. All original code has been deposited at Zenodo and is publicly available at https://doi.org/10.5281/zenodo.21498194.
