# Supplementary material for: Region-specific weighting of sensory intensity and reward prediction error by dopamine signals
Source: iScience. 2026 Aug 14;29(9):117130. doi: 10.1016/j.isci.2026.117130 (PMC13503106; doi:10.1016/j.isci.2026.117130)
Supplement: Supplementary file 1 [file mmc1.pdf]

iScience, Volume *29*

Supplemental information

Region-specific weighting of sensory intensity and reward prediction error by dopamine signals

Sarah-Julie Bouchard, Joël Boutin, Martin Lévesque, and Vincent Breton-Provencher

Supplemental information:

Figures S1-S6 &Tables S1-S4

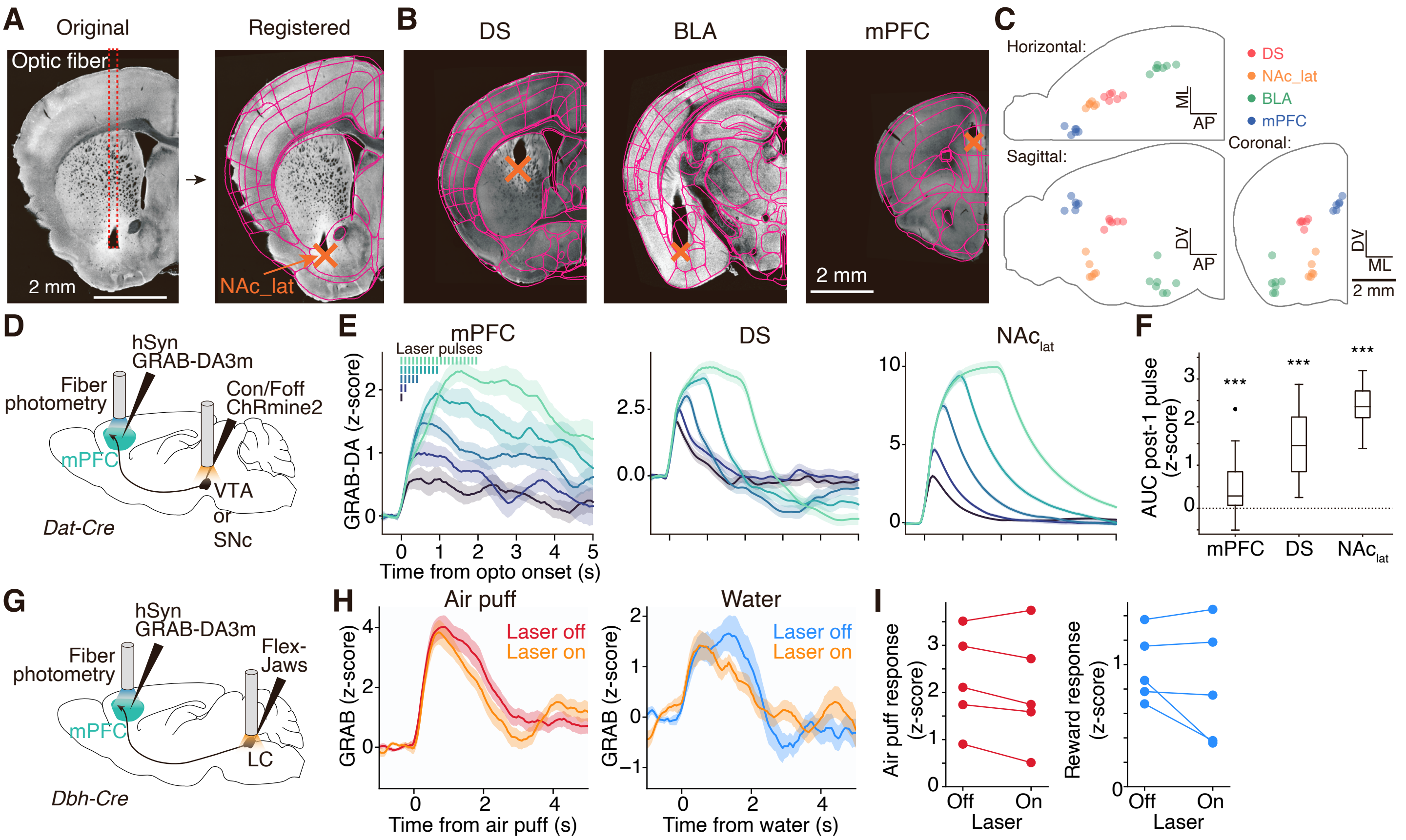

Figure S1. Validation of fiber photometry for measuring dopamine in distinct brain regions, related to Figure 1. (A) Left: A representative coronal slice showing fiber tip locations (red dashed lines) in the NAc$_{lat}$. Scale bar, 2 mm. Right: Slice alignment to the Allen Brain Atlas using point registration (atlas outlines in magenta). **(B)** Examples of fiber placements aligned to the Allen Brain reference atlas (outlined in magenta). Orange X marks indicate the locations of fiber tips. Scale bar: 2 mm. **(C)** Anatomical locations of all fiber tips used for the analysis of multisite dopamine signals (N = 25 recording sites). Scale bar: 2 mm. **(D)** Surgical strategy for optogenetic activation of dopamine-expressing neurons in the ventral tegmental area (VTA) or substantia nigra pars compacta (SNc). **(E)** Mean GRAB-DA response to different patterns of light stimulation in DA neurons expressing the opsin ChRmine2.0. **(F)** Mean response to a single 10-ms pulse of light, calculated within a 1-s post-stimulus window. N = 30 trials per condition. ***: $P < 0.001$ using a Wilcoxon signed-rank test. **(G)** Surgical strategy for optogenetic silencing of noradrenaline-expressing neurons in the locus coeruleus (LC). **(H)** Session average from an example mouse showing GRAB-DA response in the mPFC to air puff or water with (laser on) or without (laser off) LC silencing. **(I)** Mean GRAB-DA response in the mPFC to air puff or water reward with or without LC silencing. N = 5 mice. Data in (C) and (G) are plotted as mean ± s.e.m.

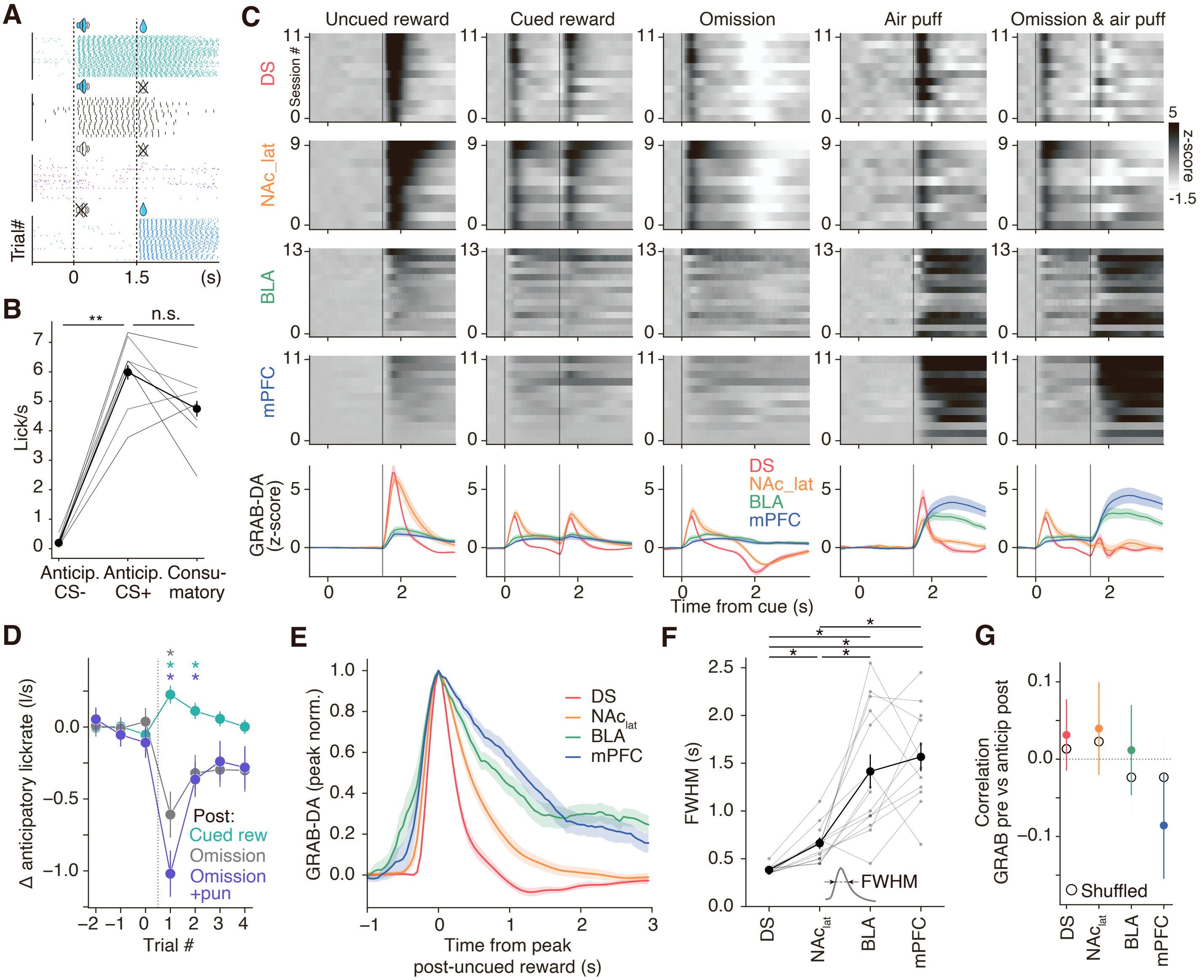

Figure S2: Mouse behavior and dopamine signals during classical conditioning paradigm, related to Figure 1 and 2. (A) Example licking behavior across all four trial types. **(B)** Average lick rate following the neutral (CS−) or conditioned (CS+) cue, or following water delivery. **$p < 0.01$, Wilcoxon signed-rank test. N = 7 mice. **(C)** Heatmaps of cue-aligned GRAB-DA signals across sessions for each region during uncued reward, cued reward, reward omission, air puff, and combined omission & air puff. Bottom panels show mean ± SEM traces for DS, NAc_{lat}, BLA, and mPFC. **(D)** Change (Δ) in anticipatory lick rate on the subsequent trials following either cued reward, reward omission, or reward omission combined with air puff. *: $p < 0.01$ using t-tests with Bonferroni correction to compare change in lick rate following a stimulus versus a shuffled sequence. N = 55 sessions. **(E)** Mean GRAB-DA responses to uncued reward for different recording targets. Responses were peak-normalized and aligned to the time of peak response. **(F)** Full width at half maximum (FWHM - see inset) of the GRAB-DA response to reward across targets. Gray lines indicate individual animals; black lines show group means ± SEM; *: $p < 0.05$ using repeated-measures ANOVA followed by Holm-corrected post-hoc tests to compare response duration across regions. (**G**) Correlation between the amplitude of GRAB-DA signal following any outcome and the anticipatory lick rate on the subsequent trials. Data are plotted separately for DA recorded in distinct locations. We did not observe a direct correlation between the amplitude of DA signal and licking behavior.

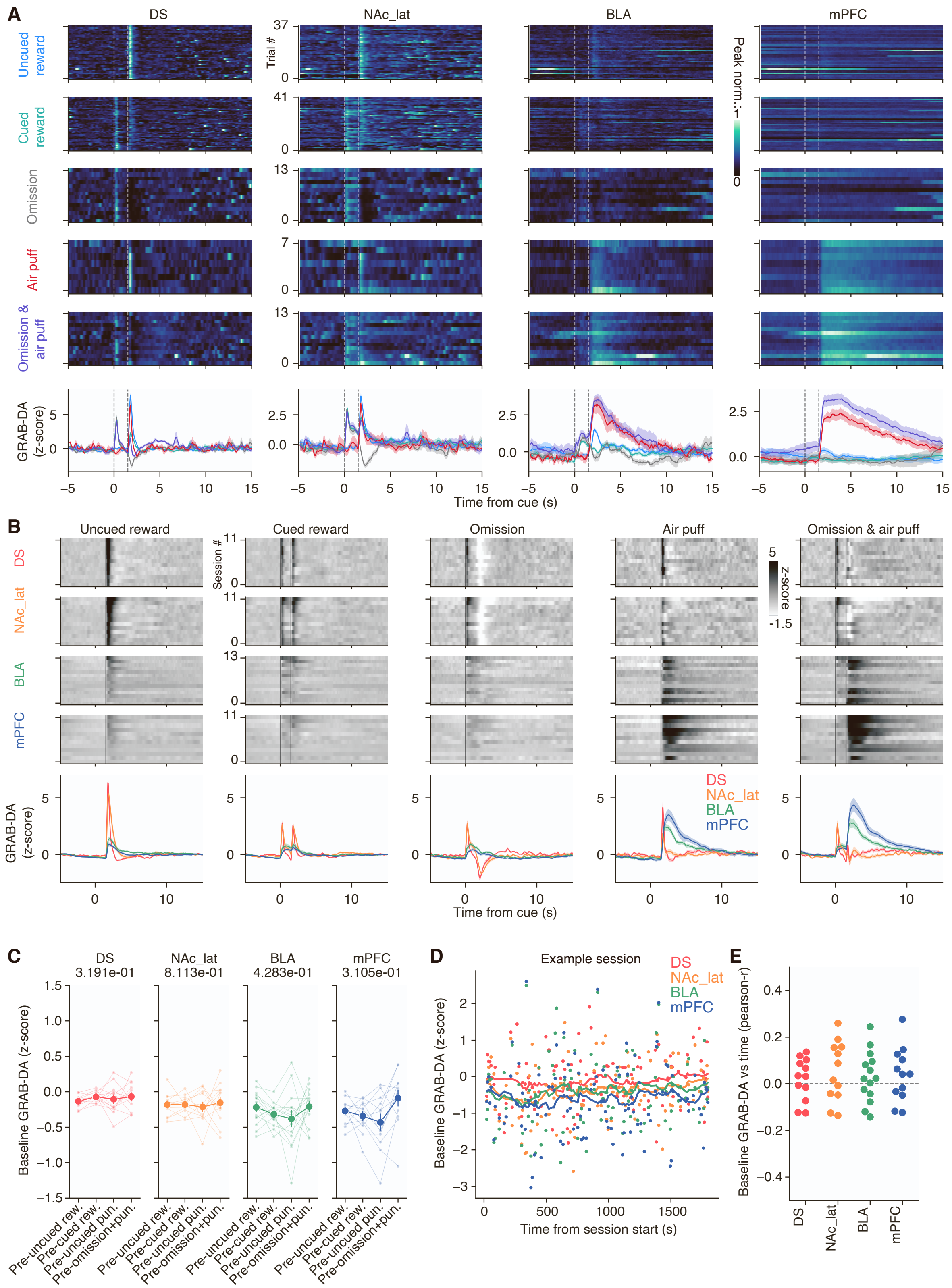

Figure S3: Baseline dopamine signals during classical conditioning paradigm, related to Figure 1 and 2. (A) Top - Raster plots of an example session showing GRAB-DA responses in four regions aligned to cue onset for each trial type. The data are from the same example shown in **Fig. 1F,G**, but are displayed without baseline subtraction and over a longer time window to illustrate the full extent of the signal across the trial. Bottom - Average GRAB-DA responses (mean ± SEM) for each trial type and region aligned to cue onset. Colors are the same as in top rasters. (**B**) Raster plots of cue-aligned GRAB-DA signals across sessions for each region during uncued reward, cued reward, reward omission, air puff, and combined omission & air puff. Bottom panels show mean ± SEM traces for DS, NAc_{lat}, BLA, and mPFC. Note that the data are the same as in **Fig. S2C**, but session averages were calculated without baseline subtraction and over a longer time window to illustrate the full extent of the signal across the trial. (**C**) Comparison of average baseline GRAB-DA signals

measured during 1s before different trial types for each region (mean ± SEM). (**D**) Baseline GRAB-DA signal measured during 1s preceding different trial types as a function of time from session start for an example session. (**E**) Correlation between baseline GRAB-DA signal and time from session start across recording locations.

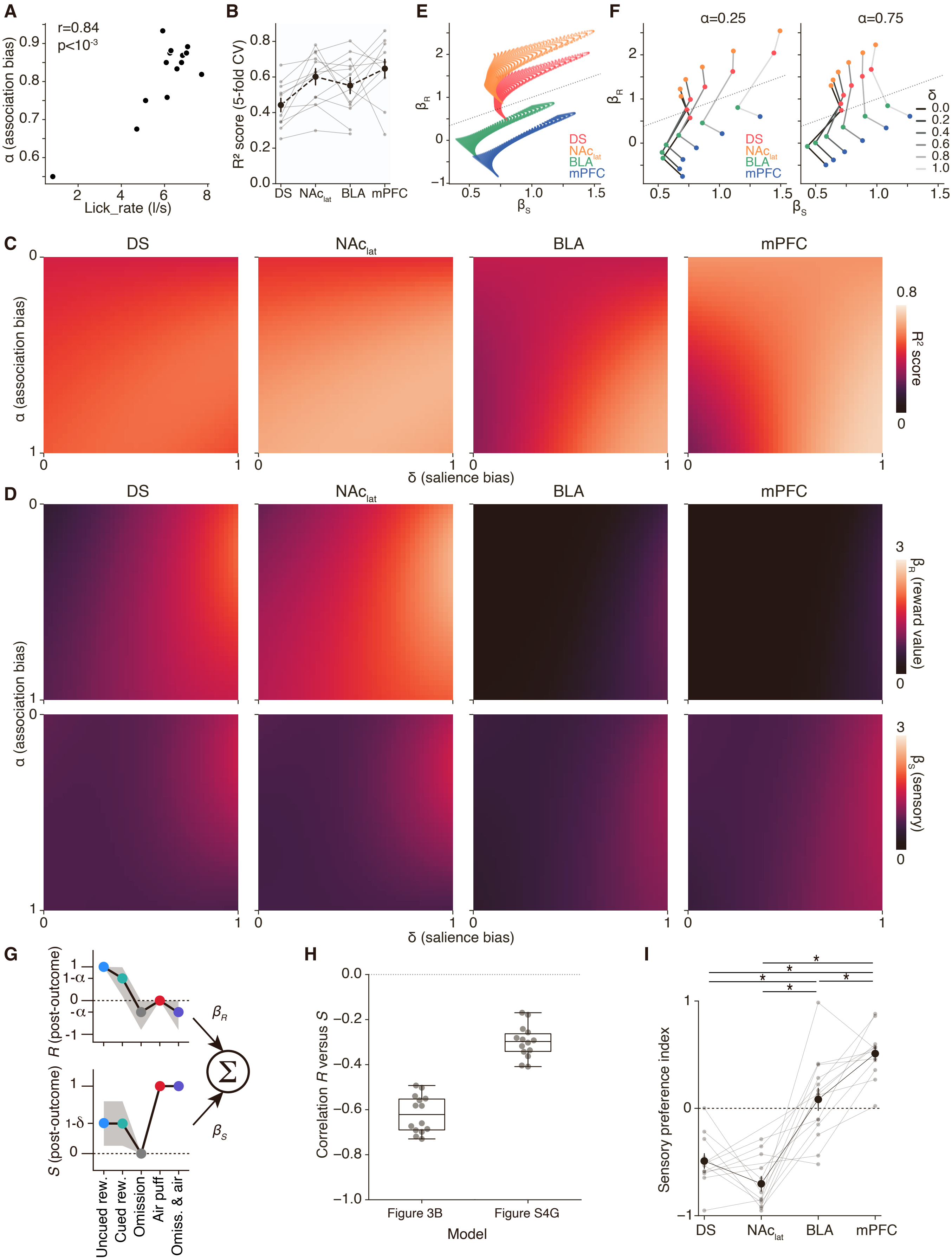

Figure S4. Impact of model parameters on constrained least-squares regression performance, related to Figure 3. (A) Association bias (α) of our model as a function of the average anticipatory lick rate after the CS+. (**B**) Prediction accuracy (r2-score R^2) of our model

across regions using 5-fold cross validation. Gray lines indicate individual animals; black lines show group means ± SEM. **(C)** Heatmaps showing the averaged model fit quality (R^2) across combinations of association bias (α) and salience bias (δ) for DS, NAc_lat, BLA, and mPFC. **(D)** Heatmaps showing estimated weights for RPE (β_R, top) and sensory (β_S, bottom) components across the same parameter space (α vs. δ) for each region averaged for all sessions. **(E)** Relationship between β_R and β_S across regions for varying α and δ values; each point represents a parameter sweep. **(F)** β_R versus β_S for two fixed α values (0.25 and 0.75) across regions. For each α, components obtained at different δ values are connected by lines. Dashed line represents the identity line in (E) and (F). (**G**) We repeated the analysis in Figure 3, but with the RPE regression set to zero for all punishment conditions. (**H**) Pearson correlation between the RPE (*R*) and sensory intensity (*S*) regressors computed session-by-session for the original model (**Fig. 3B**) and the alternative model in which the RPE regressor was set to zero for punishment conditions (G). Box and whisker plots show median, 25th–75th percentile, and minimum to maximum values; gray dots indicate individual sessions. (**I**) Sensory preference index for the model in (G). Gray lines indicate individual animals; black lines show group means ± SEM; *: $p < 0.05$ using repeated-measures ANOVA followed by Holm-corrected post-hoc tests to compare response across regions. N = 14 sessions from 7 mice.

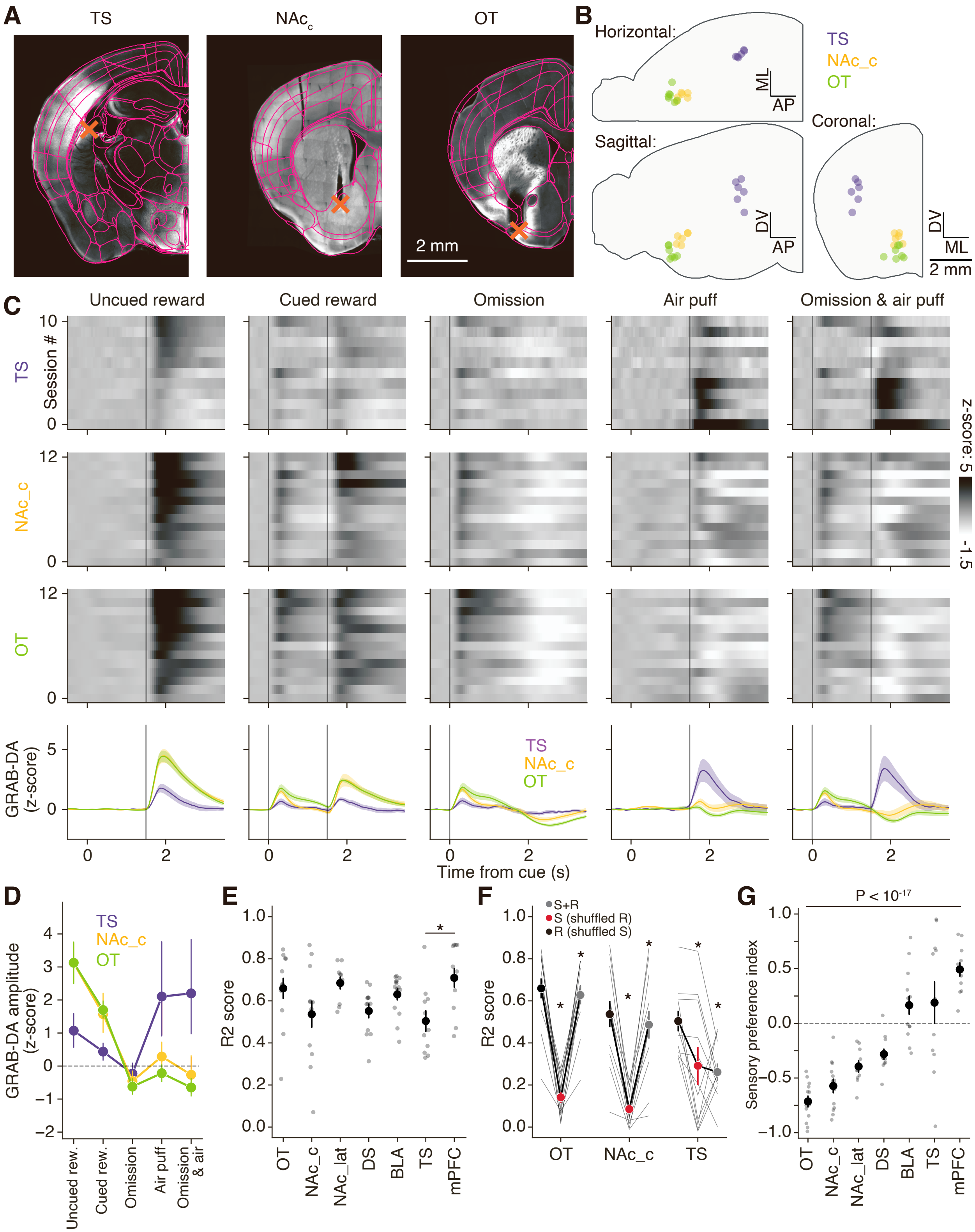

Figure S5. Dopamine dynamics in tail striatum, nucleus accumbens core, and olfactory tubercle during reward and aversive events. Related to Figure 3. (A) Representative coronal sections showing fiber placement in Tail of Striatum (TS), Nucleus Accumbens core (NAc_c), and Olfactory Tubercle (OT). Pink outlines indicate anatomical boundaries. **(B)** Location of recording sites plotted on horizontal, sagittal, and coronal planes. Colors denote regions. **(C)** Heatmaps of cue-aligned GRAB-DA signals across sessions for each region during uncued reward, cued reward, reward omission, air puff, and combined omission & air puff. Bottom panels show mean ± SEM traces for TS, NAc_c, and OT. **(D)** GRAB-DA amplitude across conditions for TS, NAc_c, and OT (mean ± SEM). **(E)** Prediction accuracy (R^2) of our model across regions. Gray dots indicate individual sessions; black lines show group means ± SEM. *: $p < 0.05$ One-way ANOVA using Tukey post-hoc **(F)** Average prediction accuracy for modeling GRAB-DA signal using the full model (gray), a model with shuffled M (red), shuffled S (black). *: $p < 0.05$ using a two-sided paired t-test with Bonferroni correction to compare full compared to partial models. **(G)** Sensory preference index across regions, showing significant heterogeneity. $P < 10^{-17}$ using one-way ANOVA. N =

2 sessions for each recording site listed in **Table S1, S4**.

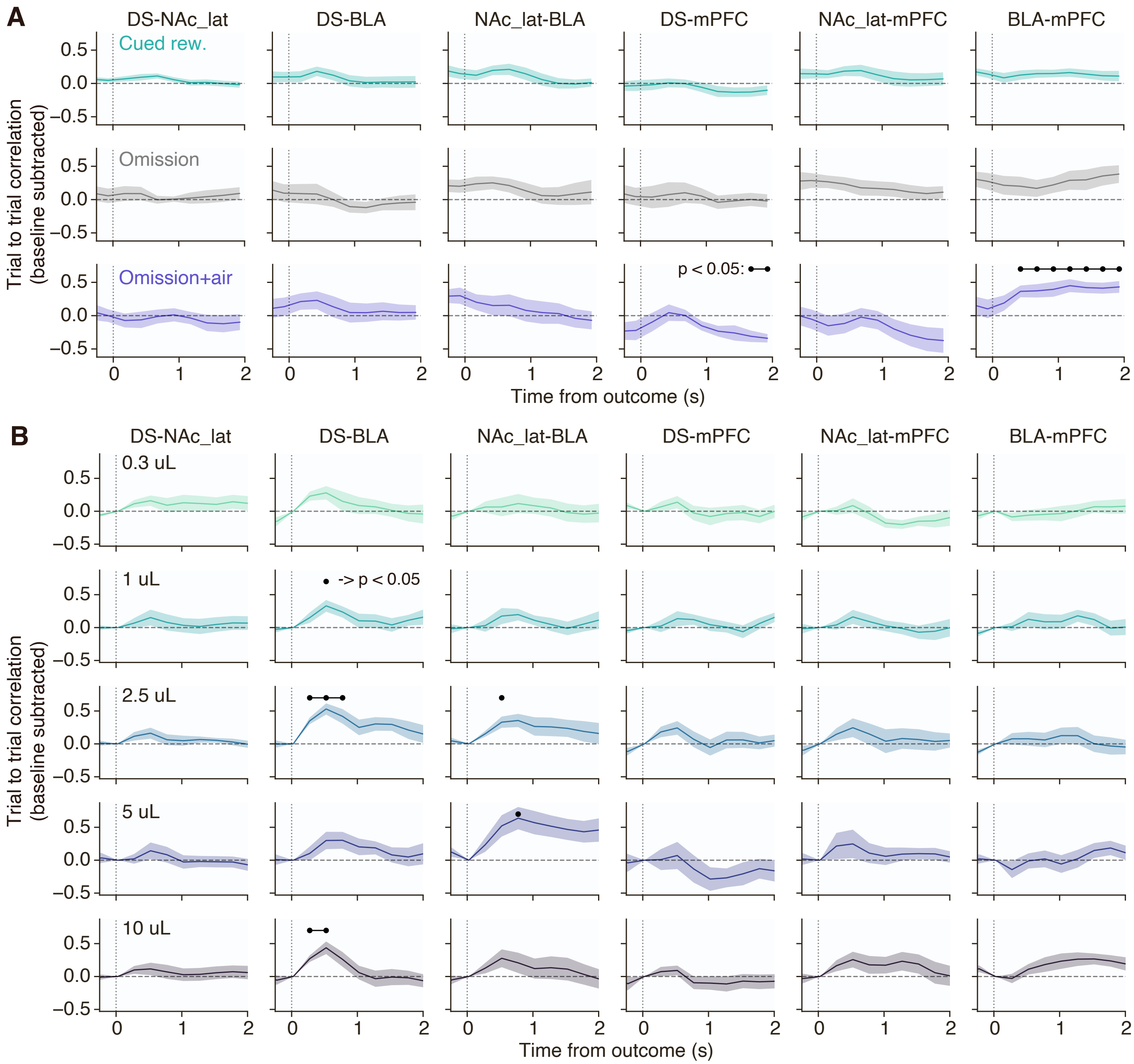

Figure S6. Time-resolved trial-to-trial correlations of dopamine signals across region pairs and task epochs. Related to figure 5. Time-resolved noise correlations for all region pairs following cued reward, reward omission, and omission with air puff (**A**); or following uncued reward of different sizes in uL (**B**). Correlations are computed for each time point and baseline-subtracted relative to the pre-stimulus period. Shaded regions indicate ± SEM across recording pairs. *: $p < 0.05$ paired t-test with Bonferroni correction comparing correlation with baseline at different timing after a stimulus. N = 10 DS-NAclat, 10 DS-mPFC, 10 NAclat-mPFC, 12 BLA-mPFC, 12 NAclat-BLA, and 12 DS-BLA recording pairs.

Site #	Animal id	Sex	Age (days)	Recording site	Coordinates (x 10 um) ML	DV	AP
1	A	M	119	BLA	243	644	696
2	A	M	119	DS	366	370	541
3	A	M	119	mPFC	519	295	329
4	A	M	119	NAc_lat	393	619	400
5	B	M	119	BLA	242	694	721
6	B	M	119	DS	383	370	514
7	B	M	119	mPFC	519	319	324
8	B	M	119	NAc_lat	403	621	390
9	C	F	97	BLA	227	649	781
10	C	F	97	DS	391	358	478
11	C	F	97	mPFC	531	289	339
12	C	F	97	NAc_lat	408	603	418
13	D	M	154	BLA	232	694	739
14	D	M	154	DS	373	407	455
15	D	M	154	mPFC	546	254	306
16	D	M	154	NAc_lat	415	607	409
17	E	M	119	BLA	243	664	690
18	E	M	119	mPFC	505	321	275
19	E	M	119	NAc_lat	407	563	388
20	F	M	97	BLA	271	641	661
21	F	M	97	DS	355	374	491
22	F	M	97	NAc_lat	425	506	372
23	G	M	154	BLA	228	523	696
24	G	M	154	DS	360	368	480
25	G	M	154	mPFC	533	284	325

Table S1: List of mice and recording sites used in the study. Age at the time of recordings. Coordinates are based on Allen Mouse Brain Common Coordinate Framework (CCFv3).

Fig #	Condition	Region A	Region B	N sessions	Mean_diff	P uncorr.	P corr.
1H	cs+ vs baseline	BLA	DS	12	-0.147	0.013	0.078
1H	cs+ vs baseline	DS	mPFC	10	0.157	0.025	0.124
1H	cs+ vs baseline	DS	NAc_lat	10	0.103	0.033	0.133
1H	cs+ vs baseline	NAc_lat	mPFC	10	0.138	0.040	0.133
1H	cs+ vs baseline	BLA	NAc_lat	12	-0.061	0.385	0.770
1H	cs+ vs baseline	BLA	mPFC	12	0.003	0.959	0.959
1H	uncued rew vs baseline	NAc_lat	mPFC	10	-0.063	0.003	0.021
1H	uncued rew vs baseline	DS	mPFC	10	-0.069	0.039	0.193
1H	uncued rew vs baseline	BLA	NAc_lat	12	0.053	0.166	0.665
1H	uncued rew vs baseline	BLA	DS	12	0.036	0.237	0.710
1H	uncued rew vs baseline	BLA	mPFC	12	-0.030	0.512	0.740
1H	uncued rew vs baseline	DS	NAc_lat	10	0.018	0.370	0.740
1H	cued rew vs baseline	BLA	DS	12	-0.139	0.011	0.059
1H	cued rew vs baseline	DS	NAc_lat	10	0.174	0.010	0.059
1H	cued rew vs baseline	DS	mPFC	10	0.153	0.026	0.104
1H	cued rew vs baseline	NAc_lat	mPFC	10	0.056	0.319	0.958
1H	cued rew vs baseline	BLA	NAc_lat	12	-0.033	0.589	1.000
1H	cued rew vs baseline	BLA	mPFC	12	-0.019	0.690	1.000
1H	omission vs baseline	DS	mPFC	10	0.595	0.000	0.000
1H	omission vs baseline	BLA	DS	12	-0.571	0.000	0.000
1H	omission vs baseline	NAc_lat	mPFC	10	0.537	0.000	0.000
1H	omission vs baseline	BLA	NAc_lat	12	-0.452	0.001	0.002
1H	omission vs baseline	DS	NAc_lat	10	0.142	0.013	0.026
1H	omission vs baseline	BLA	mPFC	12	0.003	0.972	0.972
1H	uncued pun vs baseline	NAc_lat	mPFC	10	0.301	0.004	0.022
1H	uncued pun vs baseline	DS	mPFC	10	0.190	0.021	0.103
1H	uncued pun vs baseline	BLA	NAc_lat	12	-0.194	0.054	0.216
1H	uncued pun vs baseline	BLA	DS	12	-0.120	0.103	0.309
1H	uncued pun vs baseline	BLA	mPFC	12	0.009	0.339	0.678
1H	uncued pun vs baseline	DS	NAc_lat	10	-0.051	0.456	0.678
1H	omission pun vs baseline	BLA	DS	12	-0.501	0.000	0.000
1H	omission pun vs baseline	DS	mPFC	10	0.526	0.000	0.000
1H	omission pun vs baseline	BLA	NAc_lat	12	-0.536	0.001	0.002
1H	omission pun vs baseline	NAc_lat	mPFC	10	0.619	0.001	0.002
1H	omission pun vs baseline	DS	NAc_lat	10	-0.003	0.976	1.000
1H	omission pun vs baseline	BLA	mPFC	12	0.000	0.000	0.000
2C	uncued rew vs cued rew	BLA	DS	12	0.174	0.000	0.001
2C	uncued rew vs cued rew	DS	mPFC	10	-0.255	0.000	0.001
2C	uncued rew vs cued rew	NAc_lat	mPFC	10	-0.285	0.001	0.004
2C	uncued rew vs cued rew	BLA	NAc_lat	12	0.220	0.001	0.004
2C	uncued rew vs cued rew	BLA	mPFC	12	-0.074	0.149	0.298
2C	uncued rew vs cued rew	DS	NAc_lat	10	-0.018	0.743	0.743
2C	uncued pun vs omission	NAc_lat	mPFC	10	0.118	0.016	0.099
2C	uncued pun vs omission	BLA	DS	12	0.055	0.201	1.000
2C	uncued pun vs omission	BLA	NAc_lat	12	-0.014	0.813	1.000
2C	uncued pun vs omission	BLA	mPFC	12	0.020	0.497	1.000
2C	uncued pun vs omission	DS	NAc_lat	10	-0.040	0.264	1.000
2C	uncued pun vs omission	DS	mPFC	10	0.019	0.674	1.000
2C	uncued pun vs omission pu	BLA	DS	12	0.384	0.000	0.000
2C	uncued pun vs omission pu	DS	mPFC	10	-0.465	0.000	0.000
2C	uncued pun vs omission pu	BLA	NAc_lat	12	0.347	0.000	0.000
2C	uncued pun vs omission pu	NAc_lat	mPFC	10	-0.407	0.000	0.000
2C	uncued pun vs omission pu	BLA	mPFC	12	-0.065	0.183	0.365
2C	uncued pun vs omission pu	DS	NAc_lat	10	-0.086	0.225	0.365
2C	uncued rew vs uncued pur	NAc_lat	mPFC	10	-0.861	0.000	0.000
2C	uncued rew vs uncued pur	DS	mPFC	10	-0.624	0.000	0.000
2C	uncued rew vs uncued pur	BLA	NAc_lat	12	0.573	0.000	0.001
2C	uncued rew vs uncued pur	BLA	DS	12	0.391	0.001	0.002
2C	uncued rew vs uncued pur	BLA	mPFC	12	-0.172	0.010	0.020
2C	uncued rew vs uncued pur	DS	NAc_lat	10	0.134	0.232	0.232

Table S2. Statistical results for post-hoc comparisons across brain regions, related to Figures 1H and 2C. Pairwise comparisons of GRAB-DA response amplitudes between brain regions for each trial condition. Region A and Region B indicate the two regions being compared. Mean_diff indicates the mean difference in auROC values between Region A and Region B. P uncorr. indicates the uncorrected p-value from paired t-tests. P corr. indicates the p-value after Holm correction for multiple comparisons. N sessions indicates the number of recording sessions contributing to each pairwise comparison. Comparisons are grouped by figure panel (1H and 2C) and trial condition.

Fig #	Condition	Region	t-stat	p	p_corr
1H	cs+ vs baseline	DS	6.9476	0.0000	0.0001
1H	cs+ vs baseline	NAc_lat	5.3959	0.0002	0.0009
1H	cs+ vs baseline	BLA	4.9708	0.0003	0.0010
1H	cs+ vs baseline	mPFC	5.6025	0.0002	0.0006
1H	uncued rew vs baseline	DS	8.8599	0.0000	0.0000
1H	uncued rew vs baseline	NAc_lat	9.0872	0.0000	0.0000
1H	uncued rew vs baseline	BLA	6.5148	0.0000	0.0001
1H	uncued rew vs baseline	mPFC	6.0346	0.0001	0.0003
1H	cued rew vs baseline	DS	4.7723	0.0006	0.0023
1H	cued rew vs baseline	NAc_lat	5.6032	0.0002	0.0006
1H	cued rew vs baseline	BLA	5.3128	0.0001	0.0006
1H	cued rew vs baseline	mPFC	3.9375	0.0023	0.0093
1H	omission vs baseline	DS	-7.6778	0.0000	0.0000
1H	omission vs baseline	NAc_lat	-5.4323	0.0002	0.0008
1H	omission vs baseline	BLA	4.4415	0.0007	0.0027
1H	omission vs baseline	mPFC	3.2731	0.0074	0.0297
1H	uncued pun vs baseline	DS	4.6790	0.0007	0.0027
1H	uncued pun vs baseline	NAc_lat	3.6039	0.0041	0.0166
1H	uncued pun vs baseline	BLA	7.5448	0.0000	0.0000
1H	uncued pun vs baseline	mPFC	6.0872	0.0001	0.0003
1H	omission pun vs baseline	DS	-0.4151	0.6861	2.7442
1H	omission pun vs baseline	NAc_lat	-0.3627	0.7237	2.8949
1H	omission pun vs baseline	BLA	6.4190	0.0000	0.0001
1H	omission pun vs baseline	mPFC	5.9559	0.0001	0.0004
2C	uncued rew vs cued rew	DS	8.9142	0.0000	0.0000
2C	uncued rew vs cued rew	NAc_lat	9.0371	0.0000	0.0000
2C	uncued rew vs cued rew	BLA	2.7260	0.0173	0.0693
2C	uncued rew vs cued rew	mPFC	0.7810	0.4513	1.8052
2C	uncued pun vs omission	DS	8.4430	0.0000	0.0000
2C	uncued pun vs omission	NAc_lat	9.2826	0.0000	0.0000
2C	uncued pun vs omission	BLA	6.5746	0.0000	0.0001
2C	uncued pun vs omission	mPFC	6.0470	0.0001	0.0003
2C	uncued pun vs omission pun	DS	6.6562	0.0000	0.0001
2C	uncued pun vs omission pun	NAc_lat	5.9047	0.0001	0.0004
2C	uncued pun vs omission pun	BLA	-2.0005	0.0668	0.2671
2C	uncued pun vs omission pun	mPFC	-2.8482	0.0158	0.0634
2C	uncued rew vs uncued pun	DS	3.3813	0.0061	0.0245
2C	uncued rew vs uncued pun	NAc_lat	6.1000	0.0001	0.0003
2C	uncued rew vs uncued pun	BLA	-2.7693	0.0159	0.0637
2C	uncued rew vs uncued pun	mPFC	-5.6410	0.0002	0.0006

Table S3. Statistical results for within-region comparisons of GRAB-DA response amplitudes, related to Figures 1H and 2C. Results of paired t-tests comparing GRAB-DA amplitudes between trial conditions within each brain region. Region indicates the recorded brain area. t-stat indicates the t-statistic from the paired t-test. p indicates the uncorrected p-value. p_corr indicates the p-value after Bonferroni correction for multiple comparisons. Comparisons are grouped by figure panel (1H and 2C) and trial condition.

Site #	Animal id	Sex	Age (Days)	Recording site	Coordinates (x 10 um) ML	DV	AP
26	T	M	87	NAc_c	440	560	427
27	T	M	87	TS	250	278	707
28	U	M	136	OT	451	593	362
29	U	M	136	TS	231	339	719
30	V	M	136	OT	458	591	363
31	V	M	136	TS	267	318	692
32	W	F	96	OT	443	657	370
33	W	F	96	TS	262	293	681
34	X	M	111	OT	470	643	403
35	X	M	111	TS	235	437	722
36	Y	F	111	OT	479	651	391
37	Y	F	111	TS	263	374	689
38	Z	M	126	OT	384	647	379
39	AA	M	280	NAc_c	433	593	423
40	BB	M	280	NAc_c	458	534	453
41	LL	M	117	NAc_c	435	535	453
42	MM	M	92	NAc_c	455	549	406
43	NN	F	114	NAc_c	474	586	400
44	OO	F	96	OT	440	615	365

Table S4: Additional list of mice and recording sites used in Fig. S5. Age at the time of recordings. Coordinates are based on Allen Mouse Brain Common Coordinate Framework (CCFv3).
